# DNA methylation signatures associated with cardiometabolic risk factors in children from India and The Gambia: results from the EMPHASIS study

**DOI:** 10.1186/s13148-021-01213-3

**Published:** 2022-01-09

**Authors:** Elie Antoun, Prachand Issarapu, Chiara di Gravio, Smeeta Shrestha, Modupeh Betts, Ayden Saffari, Sirazul A. Sahariah, Alagu Sankareswaran, Manisha Arumalla, Andrew M. Prentice, Caroline H. D. Fall, Matt J. Silver, Giriraj R. Chandak, Karen A. Lillycrop, the EMPHASIS study group, Sarah Kehoe, Kalyanaraman Kumaran, Ramesh D. Potdar, Sara Sajjadi, Suraj Nongmaithem, Harsha Chopra, Harshad Sane, Meera Gandhi, Stephen Owens, Landing Jarjou, Ann Prentice

**Affiliations:** 1 School of Medicine, University of Southampton, Southampton, UK; 2 Genomic Research On Complex Diseases (GRC Group), CSIR-Centre for Cellular and Molecular Biology, Hyderabad, India; 3 MRC Lifecourse Epidemiology Unit, University of Southampton, Southampton, UK; 4 School of Basic and Applied Sciences, Dayananda Sagar University, Bangalore, India; 5 MRC Unit The Gambia at the London, School of Hygiene and Tropical Medicine, Fajara, The Gambia; 6 MRC Unit The Gambia at the London, School of Hygiene and Tropical Medicine, London, UK; 7 Centre for the Study of Social Change, Mumbai, India; 8 Biological Sciences, University of Southampton, Southampton, UK

**Keywords:** EMPHASIS, DNA methylation, Epigenetics, Cardiovascular disease risk, Cardiometabolic risk factors, Early childhood risk factors

## Abstract

**Background:**

The prevalence of cardiometabolic disease (CMD) is rising globally, with environmentally induced epigenetic changes suggested to play a role. Few studies have investigated epigenetic associations with CMD risk factors in children from low- and middle-income countries. We sought to identify associations between DNA methylation (DNAm) and CMD risk factors in children from India and The Gambia.

**Results:**

Using the Illumina Infinium HumanMethylation 850 K Beadchip array, we interrogated DNAm in 293 Gambian (7–9 years) and 698 Indian (5–7 years) children. We identified differentially methylated CpGs (dmCpGs) associated with systolic blood pressure, fasting insulin, triglycerides and LDL-Cholesterol in the Gambian children; and with insulin sensitivity, insulinogenic index and HDL-Cholesterol in the Indian children. There was no overlap of the dmCpGs between the cohorts. Meta-analysis identified dmCpGs associated with insulin secretion and pulse pressure that were different from cohort-specific dmCpGs. Several differentially methylated regions were associated with diastolic blood pressure, insulin sensitivity and fasting glucose, but these did not overlap with the dmCpGs. We identified significant cis-methQTLs at three LDL-Cholesterol-associated dmCpGs in Gambians; however, methylation did not mediate genotype effects on the CMD outcomes.

**Conclusion:**

This study identified cardiometabolic biomarkers associated with differential DNAm in Indian and Gambian children. Most associations were cohort specific, potentially reflecting environmental and ethnic differences.

**Supplementary Information:**

The online version contains supplementary material available at 10.1186/s13148-021-01213-3.

## Background

Cardiometabolic disease (CMD) describes a range of conditions characterised by insulin resistance (IR), impaired glucose tolerance, dyslipidaemia and hypertension, risk factors for type 2 diabetes and cardiovascular disease (CVD). The increasing prevalence of CMDs poses a serious health burden. Although CMD is traditionally associated with high-income countries (HICs), prevalence has rapidly increased in low- and middle-income countries (LMICs) [1, 2]. Globally, the prevalence of type 2 diabetes has increased between 1980 and 2014 [3], but at higher rates in LMICs [1]. The prevalence of childhood hypertension in Central India is reported to be 6.8–7.0% [4] rising to 9.5% in Chennai [5], compared to 4% globally. Furthermore, mortality occurs earlier in LMICs, with the number of years spent living with these conditions increasing [2, 3], escalating the societal and individual health burden. The rapid rise in CMD cannot be explained solely by fixed genetic factors, but suggests that environmental factors may contribute, including a change from traditional to western diets, increased intake of processed foods, urbanisation and reduced physical activity [6, 7]. Moreover, there is substantial evidence that early life environmental exposures during critical developmental windows modulate CMD risk [8, 9]. Exposure to persistent undernutrition, poor quality diets and a high burden of infectious diseases in utero and in early childhood are suggested to induce metabolic adaptations to aid survival. However, these adaptations may be detrimental in later life, limiting metabolic capacity in response to an obesogenic environment [10]. The early onset of cardiovascular and metabolic conditions in adults from LMICs compared to HICs may reflect such adverse early life adaptations.

The environment can influence phenotype through epigenetic processes. The most widely studied epigenetic mechanism is DNA methylation (DNAm), with evidence from both human and animal studies linking environmental exposures to DNAm and metabolic changes and altered CMD risk susceptibility [11–13]. In humans, candidate gene and epigenome-wide association studies (EWAS) have identified robust associations between DNAm and CMD traits in adulthood, which have been replicated across cohorts [14–16]. However, EWAS have primarily been carried out in HIC cohorts, with limited analysis of individuals from LMICs. As DNAm is influenced by both the environment and genotype [17], the extent to which methylation markers of CMD traits from HIC can be extrapolated to LMICs is unknown. Moreover, previous EWAS have focussed on CMD-associated DNAm changes in adults. Limited studies have examined DNAm in children, where the influence of early life environmental exposures may be stronger, with the possibility to detect methylation signatures associated with sub-clinical changes in metabolic function before disease onset.

In this study, we analysed DNAm in children from the EMPHASIS study [18] (Epigenetic Mechanisms linking Pre-conceptional nutrition and Health ASsessed in India and sub-Saharan Africa; ISRCTN14266771) which includes two LMIC cohorts, one each from India and The Gambia. Previously, we investigated the effect of maternal micronutrient supplementation on DNAm in their children [19]. Here, we sought to investigate associations of DNAm with cardiometabolic risk markers in children from each cohort, and in both cohorts combined through a meta-analysis. We also examined the potential influence of genetic variants and maternal micronutrient intervention at associated loci.

## Results

### Cohort characteristics

The characteristics of the children in the two cohorts are summarised in Table 1 and stratified by sex in Additional file 1: Table S1. There were 289 Gambian children (53.6% male), with a median age of 9.0 years and 686 Indian children (55.1% male), with a median age of 5.8 years. Mean blood pressure (systolic, diastolic and pulse pressure) was generally lower in the Indian children compared to the Gambian children. The Indian children also showed lower fasting, 30-min and 120-min glucose levels during an OGTT, whereas the Gambian children showed lower fasting insulin levels. Triglyceride and LDL levels were higher in the Indian children, whereas HDL levels were higher in the Gambian children.

**Table 1:** Cohort characteristics

	The Gambia		India		*P* value
	*N*		*N*		
% Male (*N*)	289	53.6% (155)	686	55.1% (378)	*p* = 0.879
% Maternal intervention (*N*)	289	46.3% (140)	686	46.8% (321)	*p* = 0.839
Age (years)	289	9.0 (8.6–9.2)	686	5.8 (5.6–6.0)	****p < *2.2* × *10*^*–16*^
WHO weight-for-age Z-score	289	−1.37 ± 0.91	686	−1.70 ± 1.07	***p = *6.36* × *10*^*–4*^
% Underweight (*N*)	289	20.8% (60)	686	40.2% (276)	***p = *2.78* × *10*^*–8*^
WHO height-for-age *Z*-score	289	−0.72 ± 0.85	686	−1.01 ± 0.96	***p = *2.13* × *10*^*–9*^
BMI (Kg/m^2^)	289	14.4 ± 1.3	686	13.4 ± 1.4	****p < *2.2* × *10*^*–16*^
% Stunted (*N*)	289	7.6% (22)	686	15.3% (105)	**p = *0.002*
% Wasted (*N*)	289	22.5% (65)	686	37.2% (255)	***p = *2.19* × *10*^*–5*^
**Blood pressure**					
Systolic blood pressure (mmHg)	287	110.0 ± 8.2	682	92.1 ± 8.5	*p* = 0.464
Diastolic blood pressure (mmHg)	289	64.4 ± 8.1	682	56.0 ± 7.5	*p* = 0.978
Pulse pressure (mmHg)	287	45.6 ± 7.5	682	36.1 ± 6.0	*p* = 0.598
**OGTT**					
Fasting glucose (mmol/l)	280	4.81 ± 0.56	669	4.71 ± 0.50	*p* = 0.857
30-min glucose (mmol/l)	276	7.09 ± 1.40	663	6.84 ± 1.58	*p* = 0.769
120-min glucose (mmol/l)	281	5.27 ± 0.95	651	4.69 ± 0.90	*p* = 0.823
Fasting insulin (pmol/l)	287	19.2 ± 11.6	674	30.2 ± 39.9	*p* = 0.908
30-min insulin (pmol/l)	271	187.0 ± 116	656	170.1 ± 132.1	*p* = 0.146
HOMA2-S	278	0.44 ± 0.15	665	0.64 ± 0.66	*p* = 0.332
Insulinogenic index	257	8.64 ± 37.0	643	9.99 ± 51.6	*p* = 0.360
Insulinogenic index adjusted for HOMA2-S	257	21.5 ± 98.3	643	23.5 ± 130.0	*p* = 0.489
**Blood lipids**					
Triglycerides (mmol/l)	270	0.62 ± 0.22	674	0.89 ± 0.34	*p* = 0.896
HDL-C (mmol/l)	270	1.18 ± 0.29	674	1.03 ± 0.22	*p* = 0.256
LDL-C (mmol/l)	261	2.18 ± 0.59	673	2.31 ± 0.63	*p* = 0.668

Figures are either median (IQR) or mean ± sd unless otherwise specified. *P* values are from Mann–Whitney U test for continuous outcomes and Chi-squared test of independence for categorical outcomes. Stunted defined as height < -2 SD below WHO height-for-age reference mean. Wasted defined as < -2 SD below WHO reference BMI-for-age mean. WHO = World Health Organisation; BMI = Body Mass Index; OGTT = Oral Glucose Tolerance Test; HOMA2-S = Insulin Sensitivity; HDL-C = High-Density Lipoprotein Cholesterol; LDL-C = Low-Density Lipoprotein Cholesterol

### Gambian EWAS

DNA methylation was examined using the Illumina Infinium HumanMethylation 850 K Beadchip array in peripheral blood samples from the Gambian children, and robust linear regression used to identify associations between DNA methylation and concurrent cardiometabolic risk factors. A full list of significant dmCpGs (FDR < 0.05) can be found in Table 2, alongside equivalent statistics from the Indian cohort. There were no significant sex interactions with the dmCpGs identified in the Gambian children. Further details are described below.

**Table 2:** List of significant differentially methylated CpGs associated with various CMD markers

Traits	CpG ID	Genomic location (hg19)	Gambian			Indian			Gene
			Coef	*P* value	FDR	Coef	*P* value	FDR	
**Gambian dmCpGs**									
Systolic blood pressure	cg13455829	chr9:136213445	0.054	3.04 × 10^–08^	0.015	−0.002	0.747	0.997	*MED22*
	cg22671726	chr19:41305423	−0.017	3.74 × 10^–08^	0.015	0.002	0.515	0.991	*EGLN2*
	cg00368636	chr7:64734674	0.040	1.64 × 10^–07^	0.044	0.005	0.39	0.989	Intergenic
Fasting insulin	cg22388948	chr6:82460558	1.373	1.78 × 10^–08^	0.014	0.077	0.598	0.993	*FAM46A*
	cg13934266	chr15:52029533	−0.132	5.48 × 10^–08^	0.022	0.014	0.279	0.979	*LYSMD2*
Triglycerides	cg15237100	chr15:69755217	−0.439	5.32 × 10^–08^	0.031	−0.024	0.318	0.955	*RP11-279F6.1/ RP11-253M7.4*
LDL-C	cg01469688	chr9:127905934	0.132	8.74 × 10^–09^	0.004	0.041	0.019	0.916	*SCAI*
	cg06952751	chr18:21083211	0.384	9.33 × 10^–09^	0.004	0.009	0.999	0.999	*C18orf8*
	cg27229251	chr7:2680532	0.494	2.29 × 10^–08^	0.006	0.015	0.732	0.996	*TTYH3*
	cg13135286*	chr1:218302163	−4.858	5.07 × 10^–08^	0.010	0.033	0.98	0.999	Intergenic
	cg13819288	chr2:119898838	−2.259	2.20 × 10^–07^	0.035	0.163	0.392	0.987	Intergenic
	cg07988415	chr4:153291285	2.748	3.12 × 10^–07^	0.042	0.303	0.328	0.983	*FBXW7*
**Indian dmCpGs**									
HOMA2-S	cg10304969	chr1:25795613	0.154	0.387	0.986	−0.734	2.27 × 10^–08^	0.018	*TMEM57*
Insulinogenic index adjusted for HOMA2-S	cg22982428	chr2:216402618	−0.010	0.948	0.999	0.302	2.68 × 10^–08^	0.022	Intergenic
HDL-C	cg04988216	chr1:64471626	−0.223	0.190	0.974	−0.956	2.36 × 10^–08^	0.019	*ROR1*

The gene name corresponds to the nearest gene as annotated in the Illumina EPIC array manifest. CMD = cardiometabolic disease; Coef = regression beta coefficient; FDR = Benjamini–Hochberg false discovery rate; LDL-C = Low-Density Lipoprotein Cholesterol; HOMA2-S = Insulin Sensitivity; HDL-C = High-Density Lipoprotein cholesterol; dmCpG = differentially methylated CpG. Regression coefficients were calculated using beta values in the regression models for the dmCpGs to obtain interpretable coefficients

*Denotes the association between the CpG and outcome was confounded by genotype

#### Blood pressure

**Fig. 1:**
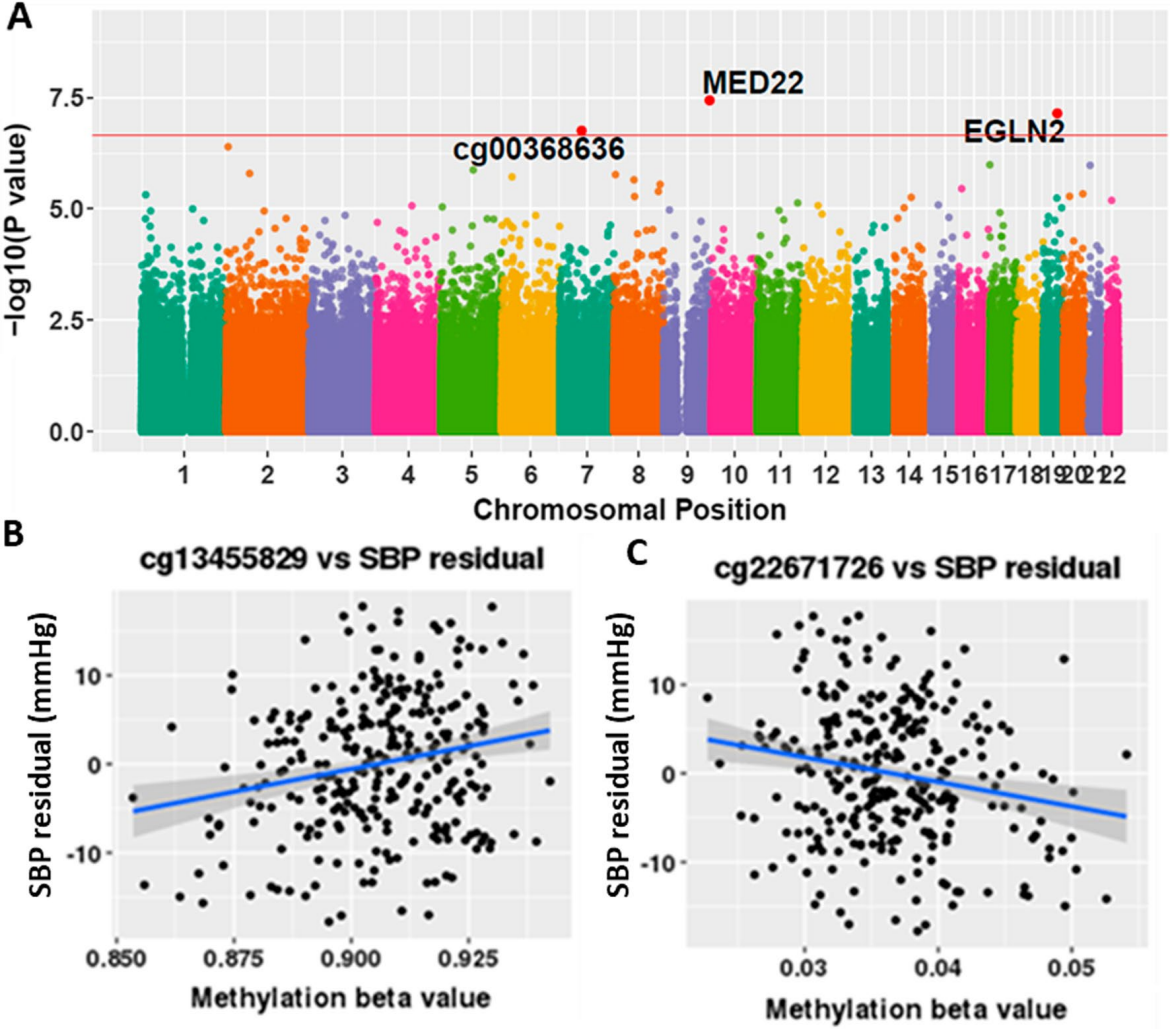
Epigenome-wide association analysis of systolic blood pressure in the Gambian cohort. **A** Manhattan plot of EWAS results with respect to systolic blood pressure (SBP) in the Gambian data. Red line indicates the Benjamini–Hochberg FDR threshold of 0.05. Significant dmCpGs (FDR < 0.05) are highlighted in red and labelled with the gene associated with them or the CpG name if intergenic. **B**–**C** Relationship between methylation beta value at **B** cg13455829 (MED22) and **C** cg22671726 (EGLN2) and SBP residual (mmHg) in 289 Gambian children. Shaded area around the regression line denotes the 95% confidence interval. EWAS, Epigenome-wide association study

There were three significant DNAm associations with systolic blood pressure (SBP) (Fig. 1a). The two most significant dmCpGs were cg13455829 in the body of the Mediator Complex Subunit 22 (*MED22*) gene (FDR = 0.015, Fig. 1b); and cg22671726 within the Egl-9 Family Hypoxia Inducible Factor 2 (*EGLN2*) gene (FDR = 0.015, Fig. 1c). A 1 mmHg SBP increase was associated with a 0.054% increase in DNAm at cg13455829 (95% CI = 0.03, 0.07) and a 0.017% decrease in methylation of cg22671726 (95% CI = −0.02, −0.01). These dmCpGs were not associated with SBP in the Indian children. There were no significant associations with diastolic blood pressure (DBP) or pulse pressure (PP).

#### Glucose levels

There were no significant associations of DNAm with children’s fasting, 30-min or 120-min glucose levels.

#### Insulin levels

There were two dmCpGs associated with fasting insulin levels: cg22388948 in the body of the Family with Sequence Similarity 46 Member A (*FAM46A)* gene (FDR = 0.014); and cg13934266 in the body of the LysM Domain Containing 2 (*LYSMD2*) gene (FDR = 0.022). A 1 pmol/l increase in fasting insulin was associated with a 1.37% increase in methylation of cg22388948 (95% CI = 0.81, 1.94) and with a 0.13% decrease in methylation of cg13934266 (95% CI = -0.18, -0.09). These dmCpGs were not associated with fasting insulin levels in the Indian children. There were no significant associations with insulin sensitivity, or insulinogenic index, unadjusted or adjusted for HOMA2-S, in the Gambian children.

#### Lipid levels

DNAm at CpG cg15237100 located in an intergenic region on chromosome 15 was associated with triglyceride levels (FDR = 0.031). A 0.1 mmol/l increase in triglycerides was associated with a 4.4% decrease in methylation of cg15237100 (95% CI = −0.63, −0.25). There were six dmCpGs associated with LDL-Cholesterol levels. The two most significant dmCpGs were cg01469688 in the promoter of the Suppressor Of Cancer Cell Invasion (*SCAI*) gene (FDR = 0.004), and cg06952751 in the promoter of the *C18orf8* gene (FDR = 0.004), with a 0.1 mmol/l increase in LDL-Cholesterol associated with a 1.3% and 3.8% increase in methylation of cg01469688 and cg06952751, respectively (cg01469688: 95% CI = 0.09, 0.18; cg06952751: 95% CI = 0.25, 0.52). There were no associations between DNAm and HDL-Cholesterol levels in the Gambian children.

### Indian EWAS

Table 2 lists all the significant dmCpGs from the Indian EWAS alongside equivalent statistics in the Gambian cohort. Some evidence of significant sex interactions was identified in the Indian children. Further details are described below.

#### Blood pressure

There were no significant associations between DNAm and SBP, DBP or PP in the Indian cohort.

#### Glucose levels

No significant associations were detected between DNAm and child’s fasting, 30-min or 120-min glucose levels.

#### Insulin levels

There were no associations between DNAm and child’s fasting insulin and insulin 30-min after an OGTT. The CpG cg10304969, in the body of the Transmembrane protein 57 (*TMEM57*) gene, was associated with insulin sensitivity (FDR = 0.018), where a 1 unit decrease in HOMA2-S was associated with a 0.73% decrease in methylation (95% CI = −0.98, −0.049). cg22982428, in an intergenic region on chromosome 2, was associated with insulinogenic index adjusted for HOMA2-S (FDR = 0.022), where a 1 unit increase was associated with a 0.3% increase in methylation (95% CI = 0.19, 0.41). These CpGs were not significant in the Gambian cohort. There were no dmCpGs associated with the insulinogenic index in the Indian cohort.

#### Lipid levels

**Fig. 2:**
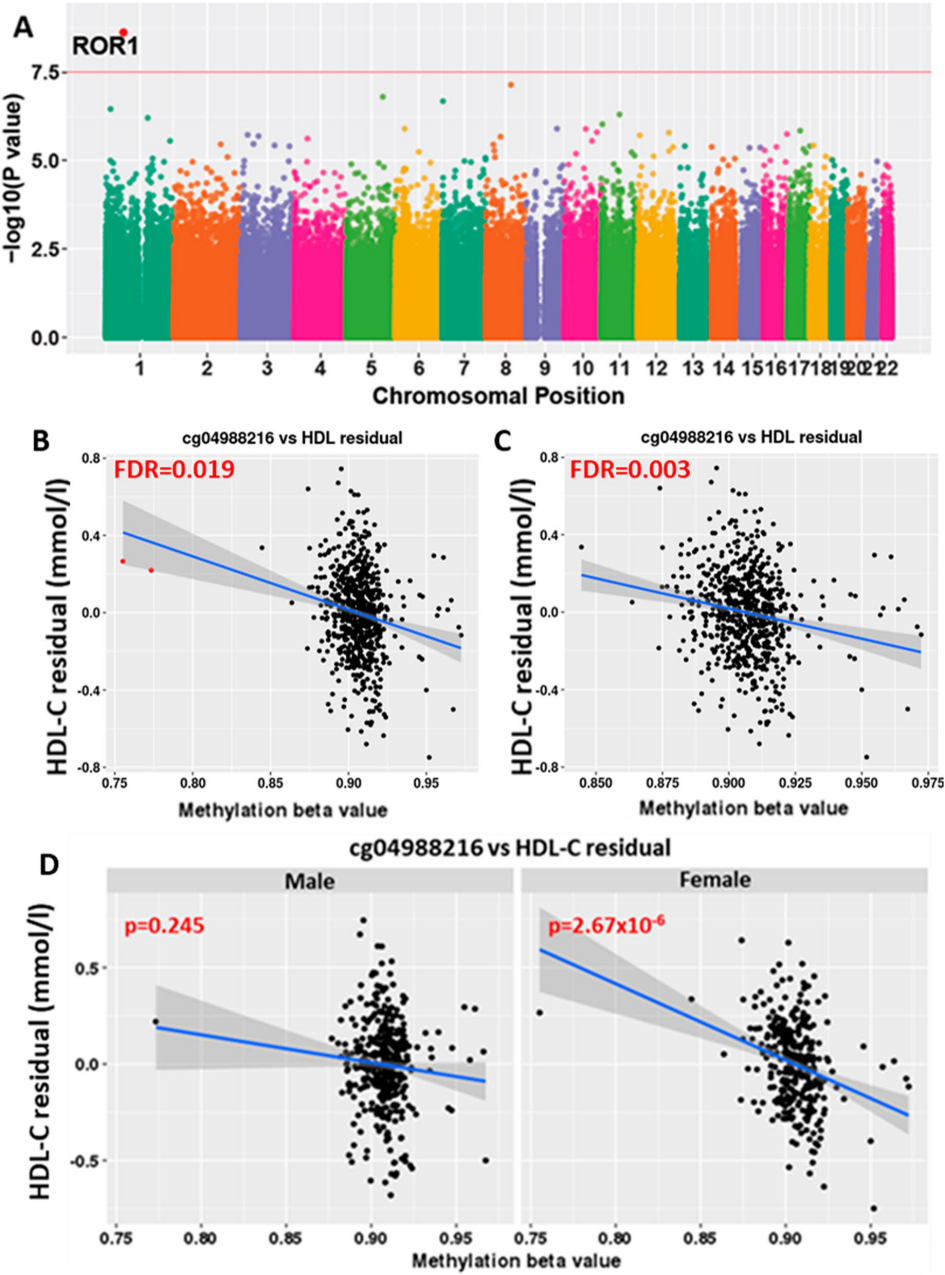
Epigenome-wide association analysis of high-density lipoprotein cholesterol in the Indian cohort. **A** Manhattan plot of EWAS results with respect to high-density lipoprotein cholesterol (HDL-C) levels in the Indian data. Red line indicates the FDR threshold of 0.05. Significant dmCpGs (FDR < 0.05) are highlighted in red and labelled with the gene associated with it. **B** Relationship between methylation beta value at cg04988216 (ROR1) and HDL-C residual (mmol/l) in 674 Indian children. Two outlier samples are highlighted in red. **C** Relationship between methylation beta value at cg04988216 (ROR1) and HDL-C residual (mmol/l) in 672 Indian children after removal of the two outlier samples highlighted in **C**. **D** Relationship between methylation at cg04988216 and HDL-C residuals (mmol/l) in the male and female children separately. Shaded area around the regression lines denotes the 95% confidence interval band. EWAS, Epigenome-wide association study

There were no significant associations with triglyceride or LDL-Cholesterol levels. One CpG was associated with HDL-Cholesterol levels (Fig. 2a); cg04988216 in the body of the Receptor Tyrosine Kinase Like Orphan Receptor 1 (*ROR1*) gene (FDR = 0.019, Fig. 2b), where a 1 mmol/l increase in HDL-C was associated with a 0.96% decrease in methylation (95% CI = −1.27, −0.64). Figure 2b suggests this association may be influenced by two outlier samples with a lower methylation beta value relative to the rest of the samples. The association was more significant (FDR = 0.003, Fig. 2c) after removal of these two samples. Furthermore, methylation at cg04988216 in *ROR1* gene showed a significant interaction with sex (*p* = 2.37 × 10^–3^, Fig. 2d), with methylation significantly associated with HDL-Cholesterol levels in the females (*p* = 2.67 × 10^–6^) but not the males (*p* = 0.24). The CpG cg04988216 was not significant in the Gambian children.

### Differentially methylated regions

To identify regional differences in DNA methylation, DMRcate was used to identify DMRs associated with the CMD markers in the children (Table 3). In the Gambian children, there was one 69 bp DMR comprising three CpGs associated with DBP (Stouffer < 0.05), located 1 kb upstream from the transcriptional start site of the Ephrin A1 (*EFNA1*) gene. One 576 bp DMR comprising nine CpGs was associated with fasting insulin levels, located in the promoter of the *C8orf31* gene. In the Indian children, one 119 bp DMR consisting of two CpGs in an intergenic region on chromosome 20 was associated with fasting glucose. Eight DMRs were significantly associated with HOMA2-S, with the top DMR comprising two CpGs located in a 13 bp intergenic region on chromosome 22. Of the DMRs identified, there were no overlaps between the cohorts. Furthermore, no DMRs were in close proximity to identified dmCpGs.

**Table 3:** List of significant differentially methylated regions associated with various CMD markers

Chromosome	Start position	End position	Width (bp)	No. of CpGs	Stouffer *p* value	Associated gene
**Gambian**						
**Systolic blood pressure**						
chr1	155,099,264	155,099,332	69	3	0.039	*EFNA1*
**Fasting insulin**						
chr8	144,120,106	144,120,681	576	9	0.010	*C8orf31*
**Indian**						
**Fasting glucose**						
chr20	49,072,540	49,072,658	119	2	0.026	Intergenic
**HOMA2-S**						
chr22	33,964,297	33,964,309	13	2	0.011	Intergenic
chr20	62,133,588	62,133,626	39	3	0.023	*RP4-697K14.3*
chr3	194,866,144	194,866,280	137	2	0.024	*RN7SL36P*
chr1	79,088,559	79,088,769	211	2	0.030	Intergenic
chr6	150,071,069	150,071,503	435	3	0.037	*PCMT1*
chr1	6,059,070	6,059,204	135	2	0.047	Intergenic
chr1	240,861,557	240,861,571	15	2	0.049	*Y_RNA.12*
chr10	1,205,222	1,205,942	721	10	0.050	*LINC00200*

Genomic coordinates are all from the hg19 build of the genome. CMD = cardiometabolic disease; bp = base pairs; HOMA2-S = Insulin Sensitivity

### Meta-analysis

**Fig. 3:**
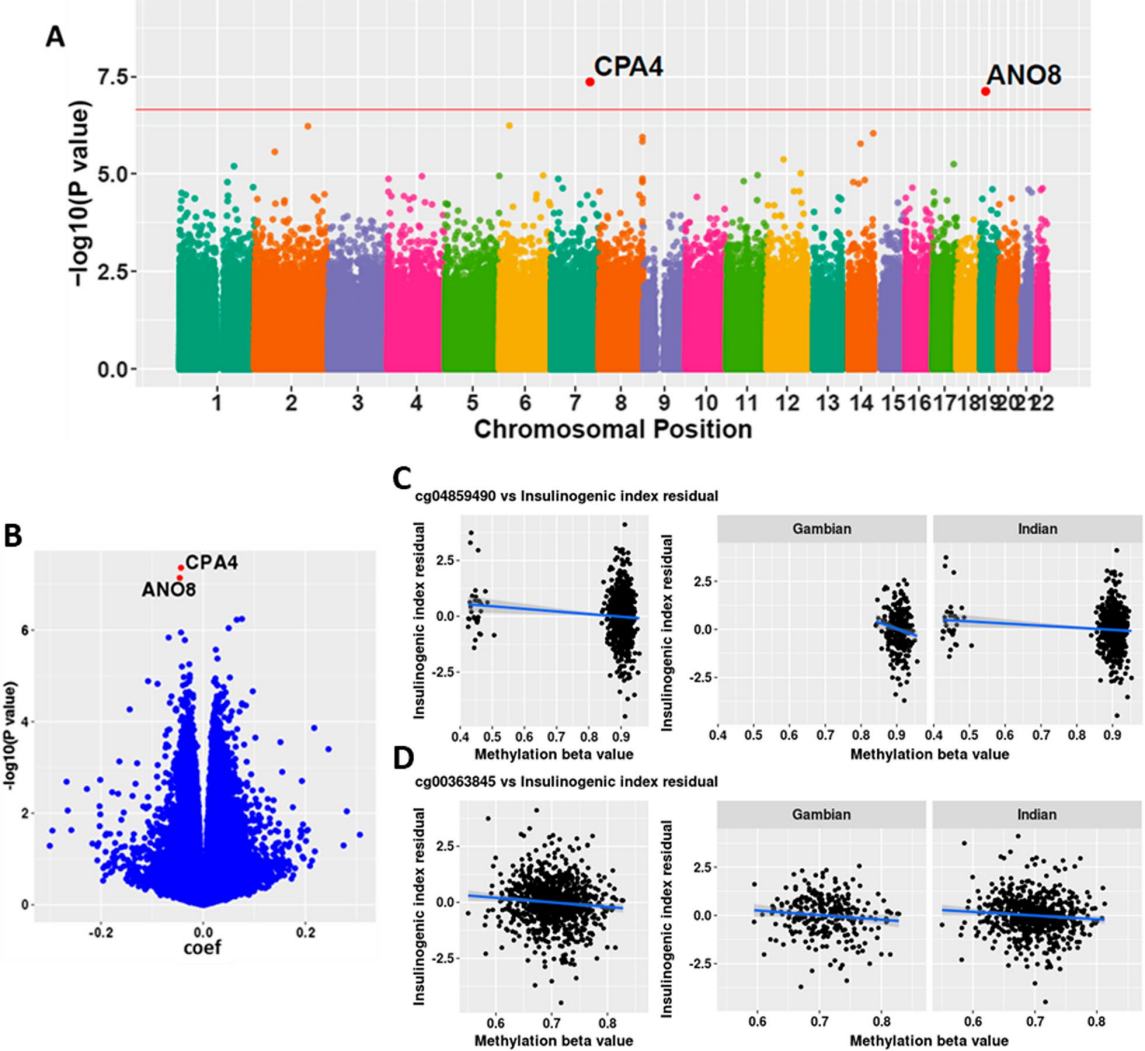
Meta-EWAS of Indian and the Gambian cohorts with respect to the Insulinogenic Index. **A** Manhattan plot of meta-analysis results with respect to the insulinogenic index. Red line indicates the adjusted *p* value threshold of 0.05. Significant dmCpGs (adjusted *p* value < 0.05) are highlighted in red and labelled with the gene associated with them. **B** Volcano plot of the meta-analysis results with respect to the insulinogenic index. Significant CpGs are highlighted in red. **C**, **D** Relationship between methylation beta value at **C** cg04859490 (CPA4) and **D** cg00363845 (GTPBP3/ANO8) and the insulinogenic index residual in combined dataset (left) and then separated by cohort (right). Shaded area around the regression lines denotes the 95% confidence interval. EWAS, Epigenome-wide association study

A meta-analysis was carried out to identify associations between DNA methylation and cardiometabolic outcomes common across both cohorts (Table 4). There were two dmCpGs significantly associated with the insulinogenic index (Fig. 3a, b). These were: cg04859490 (FDR = 0.029, HetI^2^ = 0, Fig. 3c) in intron 8 of the Carboxypeptidase A4 (*CPA4*) gene; and cg00363845 (FDR = 0.029, Het *I*^2^ = 51.7, Fig. 3d) in intron 1 of the GTP binding protein 3 (*GTPBP3*) gene. Figure 3c suggests that cg04859490 may be influenced by genotype in the Indian cohort, and we did find evidence of methQTL effects in *trans* at this locus, although these were not genome-wide significant (Additional file 1: Table S2). In a sensitivity analysis, inclusion of genotype at these nominally associated SNPs into the regression models did not influence the effect size of the association between cg04859590 and the insulinogenic index in the Indian children (Additional file 1: Table S3). One dmCpG was found to be significantly associated with pulse pressure in the meta-analysis: cg14997376 (Het *I*^2^ = 0) in exon 39 of the Citron Rho-interacting kinase (*CIT*) gene.

**Table 4:** List of significant differentially methylated CpGs associated with various CMD markers identified on meta-analysis

Gambian					Indian			Meta-analysis							Gene
CpG		Coef	*P* value	FDR	Coef	P value	FDR	Effect	SE	*P* value	FDR	Direction	HetI^2^	HetI^2^ ^*p* value^	
**Insulinogenic index**															
cg04859490		− 0.037	9.71 × 10^–02^	0.950	− 0.045	8.75 × 10^–08^	0.070	− 0.044	0.008	4.38 × 10^–08^	0.029	–	0	0.721	*CPA4*
cg00363845		− 0.062	8.13 × 10^–06^	0.284	− 0.036	5.54 × 10^–04^	0.711	− 0.046	0.009	7.30 × 10^–08^	0.029	–	51.7	0.150	*GTPBP3/ ANO8*
**Pulse pressure**															
cg14997376		0.004	6.63 × 10^–04^	0.649	0.005	3.26 × 10^–05^	0.581	0.005	0.001	4.25 × 10^–08^	0.034	+ +	0	0.700	*CIT*

Genomic coordinates are all from the hg19 build of the genome. CMD = cardiometabolic disease; Coef = regression coefficient (reported from the regressions using methylation M values); FDR = Benjamini–Hochberg false discovery rate; SE = standard error; HetI^2^, heterogeneity index

### Links to early environment and preconception nutritional supplementation

Various environmental exposures in early life have been linked to DNAm changes in children [13, 20, 21]. The mothers in both cohorts received a nutritional intervention pre- and during pregnancy [19]. Therefore, we examined whether the identified dmCpGs were associated with this intervention. None of the dmCpGs were associated with the intervention. Season of conception (SoC) is associated with DNA methylation signatures in Gambian children [22]. In the Gambian cohort, inclusion of SoC as a covariate did not influence the significance of the primary associations between CMD markers and DNAm at the dmCpGs, and no significant interaction between SoC and DNAm was observed.

### methQTL analysis

As DNA methylation at specific loci can be influenced by genotype, we used GEM to identify methylation quantitative trait loci (meQTLs) associated with the cardiometabolic dmCpGs identified in the two cohorts. For the Indian cohort, we analysed associations between the 15 CMD identified dmCpGs and 4,312,147 SNPs. No significant methQTLs were identified in *cis-* or *trans-* with a Bonferroni threshold of 2.4 × 10^–8^ and 2.8 × 10^–9^, respectively (see Methods for further details). For the Gambian cohort, we analysed associations between the 15 dmCpGs and 4,555,414 SNPs. We identified a total of 79 *cis-*methQTLs using a Bonferroni threshold of *p* = 2.4 × 10^–8^ (Additional file 1: Table S4): 44 methQTLs associated with cg00368636, 14 with cg13135286 and 21 with cg13819288. No trans-methQTLs were identified using a Bonferroni threshold *p* = 2.8 × 10^–9^. A sensitivity analysis demonstrated that effect sizes reported in the main analysis for cg00368636 and cg13819288 were not significantly affected by *cis-*methQTL effects, while the effect size for the LDL-cholesterol associated cg13135286 was significantly reduced after adjustment for the multiple *cis-*methQTLs (Additional file 1: Table S5). Additional investigation revealed that seven of the fourteen identified cg13135286-associated methQTLs were directly associated with LDL-cholesterol (Additional file 1: Table S6). We conducted further analyses to investigate the effect of these methQTLs on the reported dmCpG association (Additional file 4: Figure S3A) and observed that the methQTLs clustered into three linkage disequilibrium (LD) blocks (Additional file 1: Table S7, Additional file 4: Figure S3B, C). A mediation analysis was carried out to determine if the genotype associations with LDL-Cholesterol levels were mediated by methylation at cg13135286. We found no evidence that cg13135286 mediated the effect of the three tag SNPs (rs75332983, rs614038 and rs6659203) on LDL-Cholesterol levels (Additional file 1: Table S8).

## Discussion

In this study, we report findings from the EMPHASIS study, investigating associations between DNAm in children from two LMICs with concurrent measures of cardiometabolic risk factors. We identified novel methylation changes associated with CMD risk factors including blood pressure, insulin sensitivity and lipids at both single CpG and regional levels in the individual cohorts, and common methylation changes associated with insulin secretion and pulse pressure in a meta-analysis. These findings may provide insights into molecular pathways associated with CMD in two understudied LMIC populations.

EWAS analysis identified associations between DNAm and blood pressure. The meta-analysis identified a dmCpG within the *CIT* gene associated with pulse pressure, a measure of arterial stiffness. Furthermore, in the Gambian cohort, we found significant associations between CpGs associated with the *MED22* and *EGLN2* genes with SBP; and a DMR, comprising three CpGs, within the *EFNA1* gene associated with DBP. While MED22 has not previously been linked to vascular function, EGLN2 is involved in regulating hypoxia tolerance and apoptosis in cardiac and skeletal muscle [23]. EFNA1, an EPH receptor protein-tyrosine kinase, is highly expressed in vascular smooth muscle, triggering EPHA4 signalling and stress fibre assembly [24]. However, no studies have linked the genetic/epigenetic regulation of *EFNA1* with blood pressure. In adults, associations between DNAm and blood pressure have been reported in several cohorts [25, 26], in which trans-ethnic differences were identified [25]. However, the dmCpGs identified here did not overlap with those reported by Kazmi et al. [25] in Europeans and South Asian men or those reported in a meta-analysis of multiple African American cohorts [26].

The insulinogenic index is a measure of first-phase insulin secretion. In the meta-analysis, there were associations between CpGs within the *CPA4* and *GTPBP3* genes and insulinogenic index. Carboxypeptidase A4 (CPA4) is an exopeptidase, negatively regulating adipogenesis and downregulated during adipocyte differentiation by FGF1 [27]. CPA4 expression in adipose tissue is inversely correlated with insulin sensitivity, implicating CPA4 in maintaining local and systemic insulin sensitivity [27]. Canonically, DNA methylation across the promoter represses gene expression, while gene body methylation is generally positively associated with expression [28]. This suggests that higher methylation at this CpG may increase CPA4 expression, resulting in reduced insulin sensitivity driving increased insulin secretion in children with good pancreatic reserve, consistent with the findings reported by He et al. [27]. This is supported by the absence of association with insulin secretion adjusted for insulin sensitivity. GTPBP3 is involved in mitochondrial tRNA modification, with decreased expression associated with reduced oxygen consumption and ATP production [29]. As increased ATP is necessary for the membrane‐dependent increases in cytosolic Ca^2+^, the main trigger of insulin exocytosis [30], altered epigenetic regulation of *GTPBP3* may have downstream effects on insulin secretion. Furthermore, we found associations between DNAm and insulin measures in the individual cohorts. In the Gambian cohort, two dmCpGs were associated with fasting insulin levels, while in the Indian cohort there were associations with HOMA2-S, insulinogenic index adjusted for HOMA2-S and fasting glucose, with no overlap between the cohorts. Many of the dmCpGs and DMRs were located within intergenic regions, so their potential influence on gene expression and on insulin/glucose homeostasis is currently unclear.

Although there were no associations between DNAm and lipid levels in the meta-analysis, there were associations in the individual cohorts. Six dmCpGs were significantly associated with LDL-Cholesterol levels in the Gambian children, of which cg01469688, located in the promoter of the *SCAI* gene, was the most significant. *SCAI* is a transcriptional modulator regulating myocardin, implicated in cardiac hypertrophy [31] and hypertension [32]. There are many possible interpretations of this observation, including that LDL-Cholesterol may influence the epigenetic regulation of this cardiac transcriptional regulator, contributing to the development of CVD. Alternatively, *SCAI* transcription could influence methylation, with the dmCpG serving as a biomarker of cardiovascular stress associated with LDL-Cholesterol levels. In the Indian cohort, there were no associations of DNAm with triglycerides or LDL-Cholesterol levels. However, cg04988216 within the body of the *ROR1* gene was negatively correlated with HDL-Cholesterol levels. *ROR1* plays an essential role in skeletal and cardiac development [33]. Moreover, Sánchez-Solana et al*.* [34] have shown that inhibition of *ROR1* modulates ERK1/2 activity in mice, regulating the expression of glucose transporters 1 and 4. Decreased gene body methylation within *ROR1* may indicate decreased expression of *ROR1*, subsequently promoting increased adipogenesis in those with low HDL-Cholesterol levels, affecting susceptibility to later life metabolic disease.

In adults, previous EWAS have identified robust associations between CMD risk markers and DNAm at key genes in lipid metabolism [35]. Moreover, several of these are associated with increased CMD incidence. For example, CpGs within *CPT1A* were associated with the metabolic syndrome [36] and plasma adiponectin, a biomarker for CMD/CVD risk [37]. A recent Mendelian randomisation study has suggested a causal effect of methylation at *ABCG1* on BMI and lipid levels [14]. We did not find associations at these previously reported CpGs in our study, possibly because we measured DNAm in children without overt indications of CMD. Furthermore, the range of physiological and biochemical measures in children is smaller and presumably subject to tighter metabolic homeostasis than in adults, due to the absence of comorbidities. Moreover, the children here are from LMICs where there have been limited epigenetic studies, and differences in genotype and environment may contribute to DNAm differences associated with CMD traits.

The marked differences observed between the results in the two cohorts could reflect potential population-specific phenotypic differences. Studies have shown that Indians have greater body fat and central obesity compared to black African-Caribbean populations [38, 39]. This is reflected in higher plasma non-esterified fatty acids and triglycerides, hyperinsulinemia and IR during fasting and post-glucose challenge in the Indian population [38]. DNAm differences between the cohorts could influence, or be influenced by, the different distributions of these cardiometabolic markers, and may mark corresponding differences in IR progression.

Differences in DNAm between the populations may also result from differences in diet, environment and/or genotype. The Indian children were living in overcrowded urban slums with high levels of air pollution, which may affect the methylome [40]. In contrast, the Gambian children are from a remote rural area where the food supply is heavily season dependent [22]. However, the dmCpGs identified here were not associated with SoC or maternal pre-conceptional and pregnancy micronutrient intervention [19]. Additionally, we found limited evidence that the dmCpGs were influenced by measured genetic variation, with only three dmCpGs in the Gambian analysis having significantly associated methQTLs. We also found no evidence that methylation mediates an effect of genotype at a single CpG in Gambians where both the CpG and methQTLs were associated with LDL-Cholesterol. We note that differences in power due to the varied sample sizes of the two cohorts are unlikely to underlie the contrasting findings since effect sizes and *p* values are markedly different for dmCpGs identified in one cohort or the other (Table 2).

There are several strengths to this study. Firstly, we were able to analyse an extensive set of blood-derived markers and phenotype measures on the children, allowing detailed assessment of the relationship between DNAm and CMD markers in childhood. Secondly, investigating associations in children from two LMICs gives an opportunity to assess methylation changes in two understudied populations and for inter-cohort comparison. Although replication in HIC cohorts is possible, environmental and lifestyle differences between LMICs and HICs would be likely to confound the results. Limitations of the study include the lack of suitable cohorts from LMICs with both methylation data and CMD measures in childhood. Furthermore, DNAm was measured in peripheral blood, which has limited relevance to the aetiology of CMD traits. We also have no earlier measures of phenotypes or DNAm in the children, so cannot investigate temporal relationships. However, while we found no evidence of causal relationships, methylation changes presented here, if replicated, might serve as useful biomarkers for identifying individuals at increased risk of CMD.

## Conclusions

We carried out a comprehensive analysis of the relationship between concurrent DNA methylation in peripheral blood and measures of cardiometabolic health in children from two LMICs. We identify both cohort-specific and common associations. With further replication, identified methylation changes during early childhood may serve as biomarkers of future CMD risk and may provide insights into molecular pathways leading to CMD in later life**.**

## Methods

### Study cohorts

The EMPHASIS study [18, 19] comprises two cohorts of children born to mothers who took part in separate randomised controlled trials of nutritional supplementation before and during pregnancy. The original trials were: the Mumbai Maternal Nutrition Project (MMNP) (also known as project SARAS—ISRCTN62811278) among women living in slums in the city of Mumbai, India; and the Peri-conceptional Multiple Micronutrient Supplementation Trial (PMMST—ISRCTN13687662) among women living in rural West Kiang, The Gambia. The Mumbai children have been followed up at 5–7 years of age (“SARAS KIDS” study) and data and samples for the first 700 children studied from the per protocol group (children whose mothers started supplementation at least 3 months prior to conception) [41] have been used in the EMPHASIS study. The Gambian children were followed up aged 7–9 years in 2016; all 299 Gambian children retraced from the PMMST group were included in the study. The current investigation is a part of the Stage 2 analysis of the EMPHASIS study [18].

### Physiological and biochemical measurements

Full details of the physiological and biochemical procedures carried out are described in Additional file 5: Methods. Briefly, blood pressure was measured using an Omron HEM 7080 and Omron 705IT in the Gambian and Indian children, respectively. In both cohorts, fasting blood samples were collected. For the oral glucose tolerance test (OGTT), an oral anhydrous glucose load of 1.75 g/kg body weight was administered, after which blood samples were collected at 30 and 120 min. In the Gambian cohort, glucose and lipid concentrations were measured using the COBAS INTEGRA® 400 plus analyser (Roche Diagnostics, USA). Insulin was measured using the VITROS 350 Analyzer (Ortho Clinical Diagnostics, USA). In the Indian cohort, plasma glucose concentrations were measured using standard enzymatic methods; insulin using ELISA kits (Mercodia, Sweden); and lipids (HDL-/LDL-Cholesterol and triglycerides) using ready-to-use kits (Dialab, Austria).

### Derived variables

Insulin sensitivity (HOMA2-S) was derived from fasting glucose and insulin using the Oxford calculator (https://www.phc.ox.ac.uk/research/technology-outputs/ihoma2). Two measures of first-phase insulin secretion were derived: the Insulinogenic index [42] and the Insulinogenic index adjusted for insulin sensitivity, calculated as the residual of insulinogenic index regressed on HOMA2-S. See Additional file 5: Methods for further details.

### Processing of outcome variables

Distributions of physiological variables were checked and log-transformed if necessary. Residuals were generated for all physiological variables by adjusting for the child’s age, sex and current height and body mass index (BMI) where such associations were statistically significant and used in the final regression models. Further details are in Additional file 5: Methods.

### Epigenome-wide DNA methylation quality control (QC) and pre-processing

DNA was extracted from peripheral blood samples as previously described [19]. Epigenome-wide DNA methylation profiling was performed for a total of 698 Indian and 293 Gambian samples using the Human MethylationEPIC BeadChip platform (Illumina, USA). Full details of QC and pre-processing are described in Saffari et al. [19]. Briefly, the raw.idat files were processed in R (v3.5.2) using the Bioconductor package meffil [43]. Sex mismatches (5 Indian, 0 Gambian samples) and outlying arrays (7 Indian, 4 Gambian samples) were excluded. Probes with a low detection p value or bead numbers (1494 and 2635 probes in Indian and Gambian data, respectively), mapping to sex chromosomes and/or cross-reactive (61,523 and 61,225 probes in Indian and Gambian data, respectively) were excluded. After pre-processing and QC, this left 686 samples and 803,120 probes in the Indian cohort, and 289 samples and 802,283 probes in the Gambian cohort.

### Epigenome-wide association studies (EWAS)

#### Site-level differential methylation analysis

For EWAS analysis, robust regression models were run using *limma* (v3.38.3) [44] with methylation M values as the outcome variable due to their superior distributional properties for linear regression modelling in differential methylation analysis [45]. Models were adjusted for child’s sex, age at measurement and the first ten principle components from a methylation principle component analysis (PCA) derived from the 200,000 most variable probes to account for technical covariates and white blood cell composition [19]. The analysis was controlled for multiple testing with the Benjamini–Hochberg adjustment for false discovery rate, with a significance threshold of an FDR < 0.05. Inflation of *p* values was assessed (lambda), Quantile–Quantile (Q–Q) plots generated and *bacon* (v1.10.1) [46] used to control for genomic inflation of test statistics where lambda > 1.2.

Effect sizes for site-level analysis used methylation beta values to aid interpretation [45]. An additional investigation of interactions between methylation and sex was carried out for significant differentially methylated CpGs (dmCpGs) only.

#### Regional-level differential methylation analysis

The methylation status of adjacent CpG sites can be highly correlated, often with functional relevance and analysis of regional changes in methylation can provide increased statistical power. *DMRcate* (v1.18.0) [47] was used for the identification of differentially methylated regions (DMRs) with respect to the different measures of cardiometabolic health. DMRcate uses Stouffer’s method for combining p values for CpGs, with a Stouffer < 0.05 threshold being used as statistically significant [19].

#### Sensitivity analyses

To identify sex dependent methylation effects, we investigated potential sex-interaction effects (outcome x child sex) on the significant dmCpGs identified in the main analysis using the following regression model:

$${\text{CpG}}\_{\text{beta}}\sim {\text{Physiological}}\;{\text{Outcome}} + {\text{child}}\;{\text{sex}} + {\text{outcome}}*{\text{child}}\;{\text{sex}} + {\text{child}}\;{\text{age}} + {1}0\;{\text{principle}}\;{\text{components}}$$

Furthermore, it has previously been reported that season of conception in The Gambia influences infant’s DNA methylation and early growth. Therefore, the effect of season of conception (SoC) on the outcome-associated loci was tested by inclusion as a covariate in the main regression model and secondly, by inclusion of an interaction between DNA methylation and SoC as below:

$${\text{Outcome}}\sim {\text{CpG}}\_{\text{beta}} + {\text{SoC}} + {\text{CpG}}\_{\text{beta}}*{\text{SoC}} + {\text{child}}\;{\text{sex}} + {\text{child}}\;{\text{age}} + {1}0\;{\text{principle}}\;{\text{components}}$$

A Bonferroni-adjusted significant threshold: *p* < 2.8 × 10^–3^ (0.05/18) was used to adjust for multiple testing of the 18 dmCpGs investigated for sex interactions.

### Meta-analysis

To examine common associations across cohorts, effect size estimates from individual EWAS were meta-analysed using METAL [48] with an inverse variance weighting. Correction for inflation of both input and meta-analysis output statistics was performed using double genomic control (GC). We explored heterogeneity between the two cohorts using the *I*^2^ statistic [49, 50].

### Genotyping

SNP genotypes for 293 Gambian and 698 Indian samples were generated using the Infinium Global Screening Array-24 v1.0 BeadChip array (Illumina, USA) and imputed against a 1000 Genomes Phase 3 reference panel using IMPUTE2 (v2.3.2). Full details of QC and pre-processing can be found in Saffari et al. [19] and in Additional file 5: Methods. The final imputed data sets comprised 284 samples with 4,555,414 SNPs in the Gambian cohort, and 686 samples with 4,312,147 SNPs in the Indian cohort.

### Methylation quantitative trait loci (methQTL) analysis

methQTL analysis was carried out by the *GEM* package (v1.10.0), using an additive model [17]. The analysis was restricted to the significant dmCpGs identified in single cohort and/or meta-analyses. Separate analyses in *cis* (SNP ± 2 Mb from the CpG) and *trans* (all other SNPs) were conducted to maximise power. Significant *cis*-methQTLs were those with a Bonferroni-adjusted *p* value < 2.4 × 10^–8^, while *trans*-methQTLs were those with a Bonferroni-adjusted *p* < 2.8 × 10^–9^. Full details are described in Additional file 5: Methods.

In order to minimise non-genetic variation in the DNA methylation data, we regressed out the effect of child sex, plus the first 10 principal components (PCs) from an unsupervised PCA of the methylation data, prior to performing the methQTL analysis. The resulting methylation residuals were then rank transformed and centred to have mean 0 and variance 1 [51]. The methQTL analysis was carried out using the *GEM* package (v1.10.0) from Bioconductor which uses an additive (allelic dose) model to test for SNP-methylation associations [17]. This analysis was restricted to all 18 CpGs identified in single cohort and/or meta-analyses.

Separate analyses in *cis* (SNP ± 2 Mb from the CpG) and *trans* (all other SNPs) were conducted to maximise power. Significant *cis*-methQTLs were those with a Bonferroni-adjusted *p *value < 2.4 × 10^–8^, while *trans*-methQTLs were those with a Bonferroni-adjusted *p* < 2.8 × 10^–9^.

i. Identification of cis-methQTL

Only SNPs within + / − 2 MB of a CpG of interest were considered for this analysis. Significant *cis*-methQTL are those with an association p value passing a Bonferroni-adjusted significance threshold of *p* = 0.05/n_SNPs/n_CpGs, where n_SNPs is the number of cis-SNPs and n_CpGs is the total number of CpGs tested (*n* = 18 CpGs).

ii. Identification of trans-methQTL

*trans*-methQTL are methQTL with an association p value passing a genome-wide Bonferroni-adjusted threshold of *p* = 5 × 10^–8^/n_CpGs that do not fall within the set of *cis-*methQTL identified in i) above.

For CpGs with significant methQTLs, we conducted a sensitivity analysis to see if methQTL effects significantly changed effect sizes for the CpG-outcome associations reported in the main EWAS analysis. We did this by repeating the main analysis with the methQTL-SNP as an additional covariate and comparing original effect size estimates with 95% confidence intervals for the effect size in the adjusted model.

We also investigated associations between identified methQTLs and the traits associated with the methQTL-associated CpG identified in the main EWAS, using an additive (allelic dose) model with methylation values transformed as above. For CpGs with significant methQTL-trait associations, we tested the potential for this association to be mediated by methylation changes at the associated CpG using the mediate() function from the *mediation* package [52]*.* Confidence intervals for direct and indirect (mediated) effects were calculated using a nonparametric bootstrap with 1000 simulations.

## Supplementary Information

**Additional file 1**. Online supplementary tables.

**Additional file 2**:** Supplementary figure 1**. Q-Q plots of Indian EWAS.

**Additional file 3: Supplementary figure 2**. Q-Q plots of Gambian EWAS.

**Additional file 4**:** Supplementary figure 3**. Effect of methQTLs on dmCpGs associations.

**Additional file 5**. Online supplementary methods.

## Data Availability

EPIC data have been placed on the server of the CSIR—Centre for Cellular and Molecular Biology in Hyderabad and can be accessed with permission from the authors. Bespoke code written for the analysis is available at https://github.com/asaffa/EMPHASIS.

## Abbreviations

CMD: Cardiometabolic disease
CVD: Cardiovascular disease
LMIC: Low- and middle-income countries
HIC: High-income countries
DNAm: DNA methylation
dmCpG: Differentially methylated CpG
DMR: Differentially methylated region
SBP: Systolic blood pressure
DBP: Diastolic blood pressure
PP: Pulse pressure
HDL-C: High-density lipoprotein cholesterol
LDL-C: Low-density lipoprotein cholesterol
methQTL: Methylation quantitative trait loci
FDR: False discovery rate
EWAS: Epigenome-wide association study
