# Supplementary figures and images for: DNA methylation signatures associated with cardiometabolic risk factors in children from India and The Gambia: results from the EMPHASIS study
Source: Clin Epigenetics. 2022 Jan 9;14:6. doi: 10.1186/s13148-021-01213-3 (PMC8744249; doi:10.1186/s13148-021-01213-3)

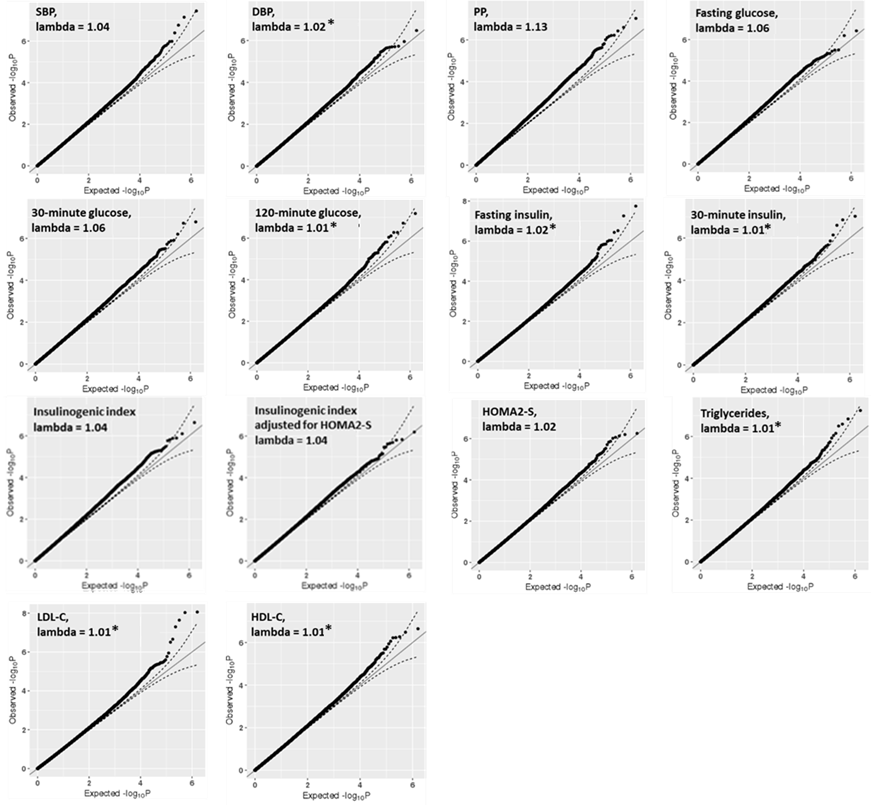

Supplement: Supplementary file 2 — Additional file 2: Supplementary figure 1. Q-Q plots of Indian EWAS. [file 13148_2021_1213_MOESM2_ESM.tif]

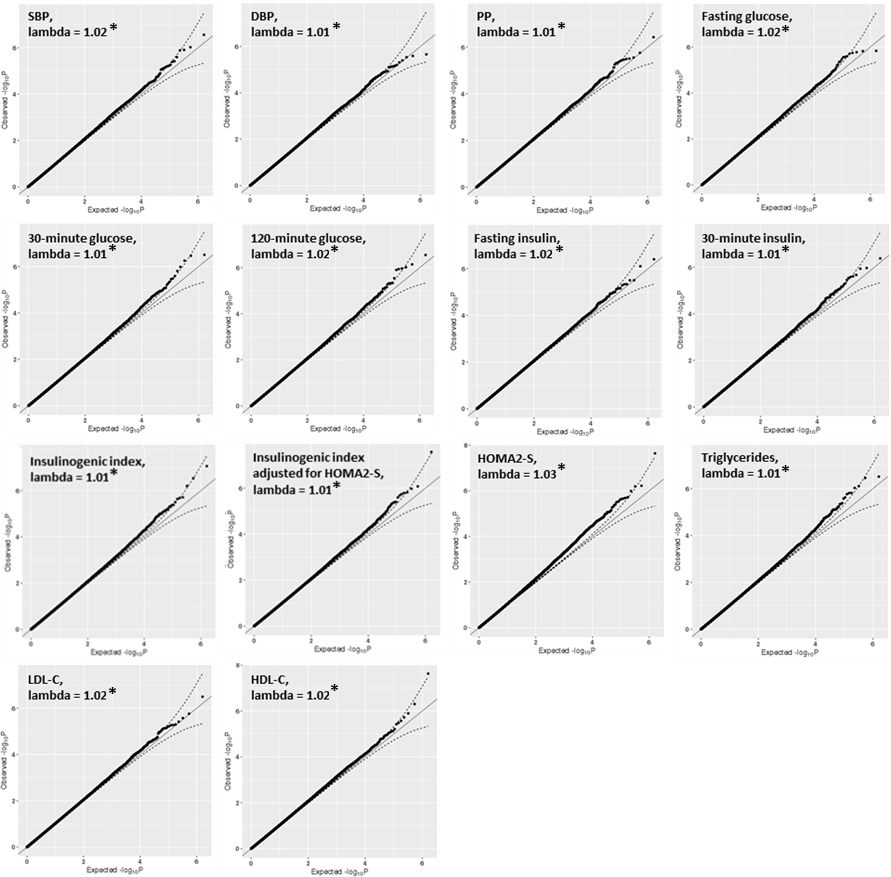

Supplement: Supplementary file 3 — Additional file 3: Supplementary figure 2. Q-Q plots of Gambian EWAS. [file 13148_2021_1213_MOESM3_ESM.tif]

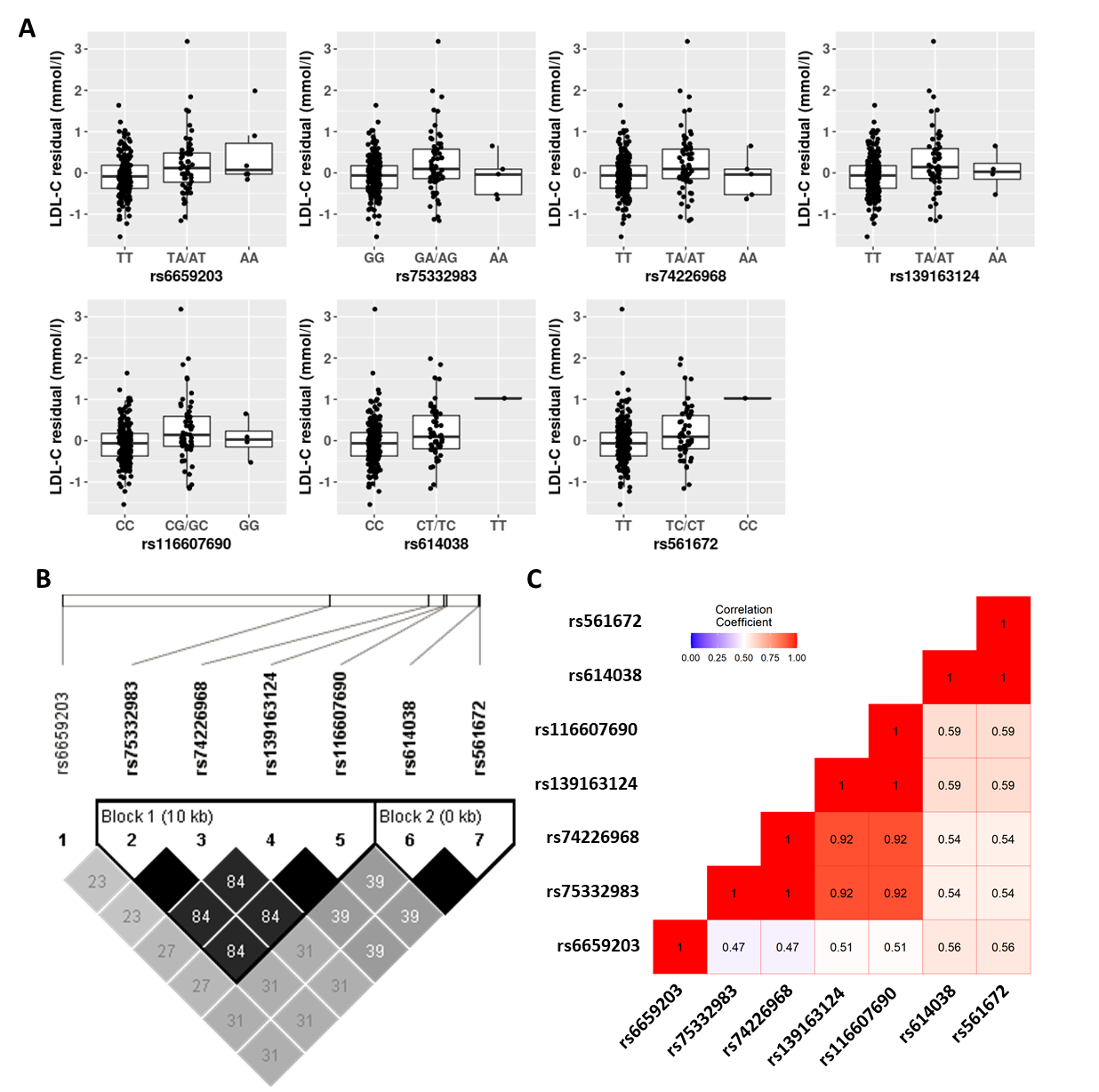

Supplement: Supplementary file 4 — Additional file 4: Supplementary figure 3. Effect of methQTLs on dmCpGs associations. [file 13148_2021_1213_MOESM4_ESM.tif]
