# Supplementary material for: DNA methylation signatures associated with cardiometabolic risk factors in children from India and The Gambia: results from the EMPHASIS study
Source: Clin Epigenetics. 2022 Jan 9;14:6. doi: 10.1186/s13148-021-01213-3 (PMC8744249; doi:10.1186/s13148-021-01213-3)
Supplement: Supplementary file 5 — Additional file 5. Online supplementary methods. [file 13148_2021_1213_MOESM5_ESM.docx]

**Online supplementary material**

**Methods**

**Blood pressure**

In the Indian cohort, systolic and diastolic blood pressures were measured using an Omron 705IT digital monitor. In the Gambian cohort, systolic and diastolic pressures were measured using an Omron HEM 7080. In both cohorts, three measurements were made after the child had been seated at rest for 5 minutes, removing and re-applying the cuff between measurements. Most were measured using the smallest child cuff (for mid-upper arm circumference (MUAC) up to 22cm); a few were measured using the next size (for MUAC up to 32 cm). The average of the three measurements was used in the analysis.

**Oral Glucose Tolerance Test (OGTT)**

In both the Gambian and Indian cohorts, fasting, baseline blood samples were collected. For the OGTT, the children were given an oral anhydrous glucose load of 1.75g/kg body weight over a period of 5 minutes, after which blood samples were collected at 30 and 120 minutes after the glucose load.

**Biochemistry**

In the Indian cohort, plasma glucose concentrations were measured in a commercial laboratory (Dr Dharap’s Laboratory, Dadar, Mumbai) using standard enzymatic methods, on the day of collection. Insulin and lipids were measured at the end of the study, using samples stored at -80°C, in the laboratory of the Diabetes Research Unit, KEM Hospital, Pune. Insulin was measured using ELISA kits (Mercodia AB, SE-754 50 Uppsala, Sweden). Lipids were measured on an automated biochemistry analyser using ready-to-use kits (Dialab, Wiener Neudorf, Austria). LDL-cholesterol was measured by a 2-step enzymatic selective protection method. HDL-cholesterol was measured using a homogeneous method without centrifugation steps; antibodies against human lipoproteins form antigen-antibody complexes with LDL, VLDL and chylomicrons in a way that only HDL-cholesterol is selectively determined by an enzymatic measurement. Triglycerides were measured using standard enzymatic kits. Inter- and intra-batch CVs for all three lipid measurements were <5%.

In the Gambian cohort, all biochemical assays were measured from the serum/plasma samples of participants using automated analysers. The COBAS INTEGRA® 400 plus analyser (Roche Diagnostics, Indianapolis, IN) was used to measure glucose concentration and lipids profiles (Cholesterol, HDL, LDL, and Triglycerides). The VITROS 350 Analyzer (Ortho Clinical Diagnostics, USA) was used to measure concentration of insulin. A daily quality control was performed on the equipment to minimize variability of results generated on different days.

**Derived variables**

Insulin sensitivity (HOMA2-S) was derived from fasting glucose and insulin values using the Oxford calculator (https://www.phc.ox.ac.uk/research/technology-outputs/ihoma2). Two measures of first-phase insulin secretion were derived: the Insulinogenic index, calculated as ln(Insulin(30-min/fasting)/Glucose(30-min/fasting)) (1, 2), and the Insulinogenic index adjusted for insulin sensitivity, calculated as the residual of insulinogenic index regressed on HOMA-S, since the insulinogenic index can increase with insulin resistance.

**Processing of outcome variables**

Physiological variables were checked for distribution and log-transformed if necessary (triglycerides and insulin). As the cardiometabolic outcomes tend to vary with age, sex and adiposity, and sometimes by height (notably blood pressure), the outcomes were adjusted for age, sex, BMI and height where such associations were statistically significant. Interactions between adjustor variables were checked and interaction terms were included if any were statistically significant (p<0.05). Adjustments were made in one step if there were no interactions (i.e. age and sex were included in a single model with the outcome to derive the residuals), but if there were age*sex interactions, age adjustments were made for males and females separately.

**Genotyping**

Genome-wide SNP genotypes for 698 Indian and 293 Gambian samples were generated using the Infinium Global Screening Array-24 v1.0 Beadchip (GSA) array (Illumina, U.S). Full details of QC and pre-processing can be found in Saffari et al. (3). Briefly, processing of the data was performed using the GenomeStudio genotyping module (Illumina, California, US). Samples with call rates <95% were excluded from downstream analysis. The data set was pre-phased for imputation using SHAPEITv2 (4) and imputation was done by IMPUTE2 (version 2.3.2) using 1000 genome phase 3 reference panel (5). SNPs with an imputation quality threshold > 0.9 were retained. Prior to mQTL analysis, the genotype data underwent further processing using PLINK (version 1.9) (6) and SNPs were filtered based on Hardy Weinberg Equilibrium (HWE) p-value < 0.00001, minor allele frequency (MAF) < 10%, and genotype missingness > 10%. The final imputed data sets comprised 686 samples with 4,312,147 SNPs in the Indian cohort and 284 samples with 4,555,414 SNPs in the Gambian cohort.

**References**

1. Wareham NJ, Phillips DI, Byrne CD, Hales CN. The 30 minute insulin incremental response in an oral glucose tolerance test as a measure of insulin secretion. Diabet Med. 1995;12(10):931.

2. Phillips DI, Clark PM, Hales CN, Osmond C. Understanding oral glucose tolerance: comparison of glucose or insulin measurements during the oral glucose tolerance test with specific measurements of insulin resistance and insulin secretion. Diabet Med. 1994;11(3):286-92.

3. Saffari A, Shrestha S, Issarapu P, Sajjadi S, Betts M, Sahariah SA, et al. Effect of maternal preconceptional and pregnancy micronutrient interventions on children's DNA methylation: Findings from the EMPHASIS study. Am J Clin Nutr. 2020;112(4):1099-113.

4. Delaneau O, Marchini J, Genomes Project C, Genomes Project C. Integrating sequence and array data to create an improved 1000 Genomes Project haplotype reference panel. Nat Commun. 2014;5:3934.

5. Genomes Project C, Auton A, Brooks LD, Durbin RM, Garrison EP, Kang HM, et al. A global reference for human genetic variation. Nature. 2015;526(7571):68-74.

6. Chang CC, Chow CC, Tellier LC, Vattikuti S, Purcell SM, Lee JJ. Second-generation PLINK: rising to the challenge of larger and richer datasets. Gigascience. 2015;4:7.

**Supplementary tables:**

**Supplementary Table 1: Cohort characteristics stratified by sex**

|  | **The Gambia** | | **India** | |
| --- | --- | --- | --- | --- |
|  | **Male** | **Female** | **Male** | **Female** |
| Maternal Intervention | 58.7% | 47.0% | 49.2% | 44.6% |
| Age (years) | 9.0 (8.6-9.2) | 9.1 (8.6-9.2) | 5.7 (5.6-6.0) | 5.8 (5.6-6.0) |
| WHO weight-for-age Z-score | -1.38±0.93 | -1.35±0.91 | -1.72±0.98 | -1.76±1.06 |
| Underweight | 21.9% | 21.5% | 39.7% | 41.5% |
| WHO height-for-age Z-score | -0.81±0.89 | -0.79±0.83 | -0.98±0.89 | -1.06±1.04 |
| BMI | 14.4±1.19 | 14.4±1.34 | 13.2±1.36 | 13.5±1.37 |
| Stunted | 9.03% | 7.95% | 13.3% | 17.9% |
| Wasted | 21.3% | 26.5% | 36.6% | 37.1% |
| **Blood pressure** | | | | |
| Systolic blood pressure (mmHg) | 109±7.97 | 111±8.70 | 91.5±8.67 | 92.9±8.40 |
| Diastolic blood pressure (mmHg) | 63.3±7.50 | 66.0±8.47 | 56.2±7.26 | 56.0±7.79 |
| Pulse pressure (mmHg) | 45.6±7.84 | 45.1±7.18 | 35.7±6.18 | 51.5±10.8 |
| **OGTT** | | | | |
| Fasting glucose | 4.89±0.56 | 4.73±0.55 | 4.65±0.51 | 4.77±0.49 |
| 30-minute glucose | 7.01±1.43 | 7.25±1.35 | 6.87±1.57 | 6.77±1.56 |
| 120-minute glucose | 5.18±0.92 | 5.37±0.97 | 4.74±0.93 | 4.62±0.84 |
| Fasting insulin (pmol/l) | 18.2±12.1 | 20.3±11.0 | 30.1±38.8 | 28.4±37.4 |
| 30-minute insulin (pmol/l) | 162±103 | 218±126 | 170±127 | 170±137 |
| HOMA2-S | 0.44±0.16 | 0.45±0.15 | 0.63±0.63 | 0.62±0.64 |
| Insulinogenic index | 11.7±47.3 | 4.57±12.9 | 12.8±61.5 | 8.97±40.2 |
| Insulinogenic index adjusted for HOMA2-S | 29.3±126 | 11.2±32.1 | 29.5±152 | 20.7±103 |
| **Blood lipids** | | | | |
| Triglycerides (mmol/l) | 0.60±0.21 | 0.65±0.24 | 0.92±0.35 | 0.88±0.34 |
| HDL-C (mmol/l) | 1.20±0.28 | 1.16±0.33 | 1.02±0.23 | 1.03±0.23 |
| LDL-C (mmol/l) | 2.06±0.59 | 2.32±0.58 | 2.35±0.64 | 2.26±0.61 |

Figures are either median (IQR) or mean±sd unless otherwise specified. Stunted defined as height < -2 SD below WHO height-for-age reference mean. Wasted defined as < -2 SD below WHO reference BMI-for-age mean. WHO = World Health Organisation; BMI = Body Mass Index; OGTT = Oral Glucose Tolerance Test; HOMA2-S = Insulin Sensitivity; HDL-C = High-Density Lipoprotein Cholesterol; LDL-C = Low-Density Lipoprotein Cholesterol

**Supplementary Table 2: Nominally significant methQTLs associated with cg04859490**

| **CpG** | **SNP** | **beta** | **Test statistic** | **P-value** | **FDR** | **CpG chromosome** | **CpG position** | **SNP Chromosome** | **SNP position** | **methQTL Type** | **REF** | **ALT** |
| --- | --- | --- | --- | --- | --- | --- | --- | --- | --- | --- | --- | --- |
| cg04859490 | rs11706717 | -0.2836 | -4.5988 | 5.06E-06 | 1 | chr7 | 129948349 | chr3 | 189847514 | trans | T | C |
| cg04859490 | rs13200235 | -0.3765 | -4.5972 | 5.10E-06 | 1 | chr7 | 129948349 | chr6 | 24100598 | trans | A | G |
| cg04859490 | rs62278560 | -0.2804 | -4.5357 | 6.78E-06 | 1 | chr7 | 129948349 | chr3 | 189851826 | trans | T | A |
| cg04859490 | rs62278563 | -0.2811 | -4.5394 | 6.67E-06 | 1 | chr7 | 129948349 | chr3 | 189855142 | trans | C | T |

**Supplementary Table 3: Sensitivity analysis of the effect of the 4 methQTL SNPS from supplementary table 2 on the association between cg04859590 and the insulinogenic index in the Indian children**

| **Regression model** | **Estimate** | **Std. Error** | **z value** | **Pr(>\|z\|)** | **Lower CI** | **Upper CI** |
| --- | --- | --- | --- | --- | --- | --- |
| cg04859490~secretion_residual+age+sex+PC1+PC2+PC3+PC4+PC5+PC6+PC7+PC8+PC9+PC10 | -0.2487 | 0.0489 | -5.0898 | 3.59x10^-7^ | -0.3445 | -0.1529 |
| cg04859490~secretion_residual+rs11706717+age+sex+PC1+PC2+PC3+PC4+PC5+PC6+PC7+PC8+PC9+PC10 | -0.2454 | 0.0495 | -4.9571 | 7.15x10^-7^ | -0.3424 | -0.1483 |
| cg04859490~secretion_residual+rs62278560+age+sex+PC1+PC2+PC3+PC4+PC5+PC6+PC7+PC8+PC9+PC10 | -0.2189 | 0.0488 | -4.4858 | 7.26x10^-7^ | -0.3146 | -0.1233 |
| cg04859490~secretion_residual+rs62278563+age+sex+PC1+PC2+PC3+PC4+PC5+PC6+PC7+PC8+PC9+PC10 | -0.2235 | 0.0486 | -4.5992 | 4.24x10^-6^ | -0.3187 | -0.1282 |
| cg04859490~secretion_residual+rs13200235+age+sex+PC1+PC2+PC3+PC4+PC5+PC6+PC7+PC8+PC9+PC10 | -0.2413 | 0.0493 | -4.8960 | 9.78x10^-7^ | -0.3379 | -0.1447 |

**Supplementary Table 4: *cis*-methQTL analysis for the dmCpGs identified**

| **CpG** | **SNP** | **beta** | **Test statistic** | **P-value** | **fdr** | **CpG position** | **SNP position** | **REF** | **ALT** | **ALT Frequency** | **Distance between CpG and mQTL (bp)** |
| --- | --- | --- | --- | --- | --- | --- | --- | --- | --- | --- | --- |
| cg00368636 | rs11977584 | 0.6687 | 8.8469 | 1.01x10^-16^ | 3.94x10^-11^ | chr7:64734674 | chr7:64750626 | T | C | 0.4809 | 15952 |
| cg00368636 | rs2672007 | 0.6687 | 8.8469 | 1.01x10^-16^ | 3.94x10^-11^ | chr7:64734674 | chr7:64754971 | C | T | 0.4809 | 20297 |
| cg00368636 | rs474341 | 0.6687 | 8.8469 | 1.01x10^-16^ | 3.94x10^-11^ | chr7:64734674 | chr7:64737545 | A | G | 0.4809 | 2871 |
| cg00368636 | rs517258 | 0.6687 | 8.8469 | 1.01x10^-16^ | 3.94x10^-11^ | chr7:64734674 | chr7:64743183 | G | T | 0.4809 | 8509 |
| cg00368636 | rs532409 | 0.6687 | 8.8469 | 1.01x10^-16^ | 3.94x10^-11^ | chr7:64734674 | chr7:64763167 | G | A | 0.4809 | 28493 |
| cg00368636 | rs537150 | 0.6687 | 8.8469 | 1.01x10^-16^ | 3.94x10^-11^ | chr7:64734674 | chr7:64752578 | A | T | 0.4809 | 17904 |
| cg00368636 | rs549970 | 0.6687 | 8.8469 | 1.01x10^-16^ | 3.94x10^-11^ | chr7:64734674 | chr7:64759913 | T | A | 0.4809 | 25239 |
| cg00368636 | rs589139 | 0.6687 | 8.8469 | 1.01x10^-16^ | 3.94x10^-11^ | chr7:64734674 | chr7:64762751 | C | T | 0.4809 | 28077 |
| cg00368636 | rs589171 | 0.6687 | 8.8469 | 1.01x10^-16^ | 3.94x10^-11^ | chr7:64734674 | chr7:64762769 | C | T | 0.4809 | 28095 |
| cg00368636 | rs590455 | 0.6687 | 8.8469 | 1.01x10^-16^ | 3.94x10^-11^ | chr7:64734674 | chr7:64763033 | T | A | 0.4809 | 28359 |
| cg00368636 | rs608809 | 0.6687 | 8.8469 | 1.01x10^-16^ | 3.94x10^-11^ | chr7:64734674 | chr7:64756869 | C | T | 0.4809 | 22195 |
| cg00368636 | rs635028 | 0.6687 | 8.8469 | 1.01x10^-16^ | 3.94x10^-11^ | chr7:64734674 | chr7:64741772 | T | C | 0.4809 | 7098 |
| cg00368636 | rs658606 | 0.6687 | 8.8469 | 1.01x10^-16^ | 3.94x10^-11^ | chr7:64734674 | chr7:64753427 | A | G | 0.4809 | 18753 |
| cg00368636 | rs666389 | 0.6687 | 8.8469 | 1.01x10^-16^ | 3.94x10^-11^ | chr7:64734674 | chr7:64760534 | C | T | 0.4809 | 25860 |
| cg00368636 | rs71562986 | 0.6687 | 8.8469 | 1.01x10^-16^ | 3.94x10^-11^ | chr7:64734674 | chr7:64754479 | C | T | 0.4809 | 19805 |
| cg00368636 | rs482461 | 0.6625 | 8.7062 | 2.69x10^-16^ | 1.01x10^-10^ | chr7:64734674 | chr7:64765176 | A | G | 0.4826 | 30502 |
| cg00368636 | GSA-rs7796960 | 0.6738 | 8.6111 | 5.20x10^-16^ | 1.89x10^-10^ | chr7:64734674 | chr7:64767457 | C | T | 0.4271 | 32783 |
| cg00368636 | rs10269825 | 0.6472 | 8.1329 | 1.34x10^-14^ | 4.08x10^-9^ | chr7:64734674 | chr7:64739577 | C | T | 0.4306 | 4903 |
| cg00368636 | rs4717245 | 0.6472 | 8.1329 | 1.34x10^-14^ | 4.08x10^-9^ | chr7:64734674 | chr7:64757551 | T | A | 0.4306 | 22877 |
| cg00368636 | rs9638427 | 0.6472 | 8.1329 | 1.34x10^-14^ | 4.08x10^-9^ | chr7:64734674 | chr7:64762034 | G | A | 0.4306 | 27360 |
| cg00368636 | rs10214965 | 0.6354 | 7.9790 | 3.73x10^-14^ | 1.07x10^-8^ | chr7:64734674 | chr7:64738517 | T | C | 0.4288 | 3843 |
| cg00368636 | rs10214971 | 0.6354 | 7.9790 | 3.73x10^-14^ | 1.07x10^-8^ | chr7:64734674 | chr7:64738754 | T | C | 0.4288 | 4080 |
| cg00368636 | rs10231956 | 0.6354 | 7.9790 | 3.73x10^-14^ | 1.07x10^-8^ | chr7:64734674 | chr7:64762394 | G | A | 0.4288 | 27720 |
| cg00368636 | rs3846968 | 0.6354 | 7.9299 | 5.16x10^-14^ | 1.47x10^-8^ | chr7:64734674 | chr7:64733634 | G | C | 0.4271 | 1040 |
| cg00368636 | rs3846969 | 0.6354 | 7.9299 | 5.16x10^-14^ | 1.47x10^-8^ | chr7:64734674 | chr7:64733683 | G | A | 0.4271 | 991 |
| cg00368636 | rs10949984 | -0.5582 | -7.3153 | 2.68x10^-12^ | 5.89x10^-7^ | chr7:64734674 | chr7:64766993 | G | A | 0.4653 | 32319 |
| cg00368636 | rs35888355 | -0.5350 | -6.9499 | 2.54x10^-11^ | 4.92x10^-6^ | chr7:64734674 | chr7:64762875 | G | C | 0.4618 | 28201 |
| cg00368636 | rs11772443 | -0.5323 | -6.9474 | 2.58x10^-11^ | 4.99x10^-6^ | chr7:64734674 | chr7:64761275 | T | C | 0.4670 | 26601 |
| cg00368636 | rs564679 | -0.5443 | -6.8255 | 5.36x10^-11^ | 9.77x10^-6^ | chr7:64734674 | chr7:64751848 | G | T | 0.4618 | 17174 |
| cg00368636 | rs34324184 | -0.5390 | -6.6684 | 1.36x10^-10^ | 2.33x10^-5^ | chr7:64734674 | chr7:64740133 | C | CT | 0.4375 | 5459 |
| cg00368636 | rs13221773 | -0.5200 | -6.5010 | 3.61x10^-10^ | 5.89x10^-5^ | chr7:64734674 | chr7:64818970 | A | G | 0.4757 | 84296 |
| cg00368636 | rs1352081 | -0.5200 | -6.5010 | 3.61x10^-10^ | 5.89x10^-5^ | chr7:64734674 | chr7:64823281 | C | G | 0.4757 | 88607 |
| cg00368636 | rs2200482 | -0.5200 | -6.5010 | 3.61x10^-10^ | 5.89x10^-5^ | chr7:64734674 | chr7:64809442 | C | T | 0.4757 | 74768 |
| cg00368636 | rs60621462 | -0.5200 | -6.5010 | 3.61x10^-10^ | 5.89x10^-5^ | chr7:64734674 | chr7:64823601 | C | T | 0.4757 | 88927 |
| cg00368636 | rs6952160 | -0.4962 | -6.2241 | 1.75x10^-9^ | 2.59x10^-4^ | chr7:64734674 | chr7:64807962 | G | C | 0.4861 | 73288 |
| cg00368636 | rs4620170 | 0.4985 | 6.1097 | 3.31x10^-9^ | 4.73x10^-4^ | chr7:64734674 | chr7:64804120 | G | A | 0.4601 | 69446 |
| cg00368636 | rs11486715 | 0.4983 | 6.1079 | 3.34x10^-9^ | 4.77x10^-4^ | chr7:64734674 | chr7:64824571 | A | G | 0.4236 | 89897 |
| cg00368636 | rs10267906 | 0.5959 | 6.0903 | 3.68x10^-9^ | 5.24x10^-4^ | chr7:64734674 | chr7:64770187 | A | G | 0.2309 | 35513 |
| cg00368636 | rs7804725 | 0.5771 | 6.0255 | 5.25x10^-9^ | 7.26x10^-4^ | chr7:64734674 | chr7:64781894 | G | A | 0.2413 | 47220 |
| cg00368636 | rs13244870 | 0.5151 | 5.9284 | 8.92x10^-9^ | 1.12x10^-3^ | chr7:64734674 | chr7:64794391 | C | T | 0.3281 | 59717 |
| cg00368636 | rs9638439 | 0.5151 | 5.9284 | 8.92x10^-9^ | 1.12x10^-3^ | chr7:64734674 | chr7:64800432 | C | T | 0.3281 | 65758 |
| cg00368636 | rs9638441 | 0.4735 | 5.8225 | 1.58x10^-8^ | 2.05 x10^-3^ | chr7:64734674 | chr7:64807261 | C | T | 0.4635 | 72587 |
| cg00368636 | rs34615570 | 0.4818 | 5.8198 | 1.60x10^-8^ | 2.07 x10^-3^ | chr7:64734674 | chr7:64734385 | TG | T | 0.4844 | 289 |
| cg00368636 | rs7796045 | -0.4708 | -5.7908 | 1.87x10^-8^ | 2.40 x10^-3^ | chr7:64734674 | chr7:64767609 | G | A | 0.3628 | 32935 |
| cg13135286 | rs74226968 | -1.5161 | -18.6218 | 5.03x10^-51^ | 3.83x10^-44^ | chr1:218302163 | chr1:218311278 | T | A | 0.1406 | 9115 |
| cg13135286 | rs75332983 | -1.5161 | -18.6218 | 5.03x10^-51^ | 3.83x10^-44^ | chr1:218302163 | chr1:218302164 | G | A | 0.1406 | 1 |
| cg13135286 | rs116607690 | -1.4839 | -15.8976 | 4.52x10^-41^ | 1.98x10^-34^ | chr1:218302163 | chr1:218313053 | C | G | 0.1215 | 10890 |
| cg13135286 | rs139163124 | -1.4839 | -15.8976 | 4.52x10^-41^ | 1.98x10^-34^ | chr1:218302163 | chr1:218312757 | T | A | 0.1215 | 10594 |
| cg13135286 | rs114957083 | -1.5025 | -14.2465 | 4.61x10^-35^ | 9.24x10^-29^ | chr1:218302163 | chr1:218299454 | A | G | 0.1076 | 2709 |
| cg13135286 | rs484354 | -1.0014 | -8.9323 | 5.53x10^-17^ | 2.20x10^-11^ | chr1:218302163 | chr1:218328472 | A | T | 0.1267 | 26309 |
| cg13135286 | exm2260003 | -1.0038 | -8.7261 | 2.34x10^-16^ | 8.80x10^-11^ | chr1:218302163 | chr1:218333601 | T | C | 0.1181 | 31438 |
| cg13135286 | rs561672 | -0.9529 | -7.6978 | 2.35x10^-13^ | 6.34x10^-8^ | chr1:218302163 | chr1:218316070 | C | T | 0.1128 | 13907 |
| cg13135286 | rs614038 | -0.9529 | -7.6978 | 2.35x10^-13^ | 6.34x10^-8^ | chr1:218302163 | chr1:218315980 | T | C | 0.1128 | 13817 |
| cg13135286 | rs74145814 | -0.6618 | -6.7566 | 8.08x10^-11^ | 1.45x10^-5^ | chr1:218302163 | chr1:218226577 | C | A | 0.1997 | 75586 |
| cg13135286 | rs511513 | -0.7617 | -6.7337 | 9.26x10^-11^ | 1.65x10^-5^ | chr1:218302163 | chr1:218332013 | T | C | 0.1476 | 29850 |
| cg13135286 | rs6663401 | -0.6209 | -6.5809 | 2.27x10^-10^ | 3.78x10^-5^ | chr1:218302163 | chr1:218230782 | G | A | 0.2170 | 71381 |
| cg13135286 | rs6659203 | -0.7378 | -6.4697 | 4.33x10^-10^ | 7.02x10^-5^ | chr1:218302163 | chr1:218277194 | T | A | 0.1302 | 24969 |
| cg13135286 | rs140539580 | -0.7839 | -6.0391 | 4.88x10^-9^ | 6.76x10^-4^ | chr1:218302163 | chr1:218248632 | AAC | A | 0.1042 | 53531 |
| cg13819288 | rs4849749 | -0.6899 | -7.2967 | 3.00x10^-12^ | 6.56x10^-7^ | chr2:119898838 | chr2:119832306 | G | A | 0.2014 | 66532 |
| cg13819288 | rs36009069 | -0.7534 | -7.0923 | 1.07x10^-11^ | 2.16x10^-6^ | chr2:119898838 | chr2:119853315 | A | G | 0.1615 | 45523 |
| cg13819288 | rs75106351 | -0.7536 | -6.6292 | 1.71x10^-10^ | 2.90x10^-5^ | chr2:119898838 | chr2:119816813 | T | C | 0.1163 | 82025 |
| cg13819288 | rs116549068 | -0.7589 | -6.5367 | 2.94x10^-10^ | 4.82x10^-5^ | chr2:119898838 | chr2:119801443 | A | C | 0.1146 | 97395 |
| cg13819288 | rs13410962 | -0.7589 | -6.5367 | 2.94x10^-10^ | 4.82x10^-5^ | chr2:119898838 | chr2:119812759 | C | T | 0.1146 | 86079 |
| cg13819288 | rs1530136 | -0.7589 | -6.5367 | 2.94x10^-10^ | 4.82x10^-5^ | chr2:119898838 | chr2:119808225 | G | A | 0.1146 | 90613 |
| cg13819288 | rs10202747 | -0.7259 | -6.4922 | 3.80x10^-10^ | 6.19x10^-5^ | chr2:119898838 | chr2:119907262 | A | G | 0.1615 | 8424 |
| cg13819288 | rs13395242 | -0.7259 | -6.4922 | 3.80x10^-10^ | 6.19x10^-5^ | chr2:119898838 | chr2:119910625 | G | C | 0.1615 | 11787 |
| cg13819288 | rs13425098 | -0.7259 | -6.4922 | 3.80x10^-10^ | 6.19x10^-5^ | chr2:119898838 | chr2:119905147 | G | A | 0.1615 | 6309 |
| cg13819288 | rs141807893 | -0.7259 | -6.4922 | 3.80x10^-10^ | 6.19x10^-5^ | chr2:119898838 | chr2:119908137 | AAG | A | 0.1615 | 9299 |
| cg13819288 | rs143070097 | -0.7259 | -6.4922 | 3.80x10^-10^ | 6.19x10^-5^ | chr2:119898838 | chr2:119911467 | TA | T | 0.1615 | 12629 |
| cg13819288 | rs7562612 | -0.7259 | -6.4922 | 3.80x10^-10^ | 6.19x10^-5^ | chr2:119898838 | chr2:119908613 | G | A | 0.1615 | 9775 |
| cg13819288 | rs9308771 | -0.7259 | -6.4922 | 3.80x10^-10^ | 6.19x10^-5^ | chr2:119898838 | chr2:119905989 | C | T | 0.1615 | 7151 |
| cg13819288 | rs9308772 | -0.7259 | -6.4922 | 3.80x10^-10^ | 6.19x10^-5^ | chr2:119898838 | chr2:119905994 | C | T | 0.1615 | 7156 |
| cg13819288 | rs9308773 | -0.7259 | -6.4922 | 3.80x10^-10^ | 6.19x10^-5^ | chr2:119898838 | chr2:119906603 | T | C | 0.1615 | 7765 |
| cg13819288 | rs9308774 | -0.7259 | -6.4922 | 3.80x10^-10^ | 6.19x10^-5^ | chr2:119898838 | chr2:119906623 | T | G | 0.1615 | 7785 |
| cg13819288 | rs1439950 | -0.6896 | -6.4524 | 4.78x10^-10^ | 7.68x10^-5^ | chr2:119898838 | chr2:119827462 | A | G | 0.1615 | 71376 |
| cg13819288 | rs745322 | -0.6896 | -6.4524 | 4.78x10^-10^ | 7.68x10^-5^ | chr2:119898838 | chr2:119825516 | T | A | 0.1615 | 73322 |
| cg13819288 | rs111838684 | -0.6919 | -6.1813 | 2.22x10^-9^ | 3.23x10^-4^ | chr2:119898838 | chr2:119903074 | A | G | 0.1649 | 4236 |
| cg13819288 | rs13409086 | -0.6919 | -6.1813 | 2.22x10^-9^ | 3.23x10^-4^ | chr2:119898838 | chr2:119903318 | A | G | 0.1649 | 4480 |
| cg13819288 | rs330773 | -0.5607 | -5.7970 | 1.81x10^-8^ | 2.33x10^-3^ | chr2:119898838 | chr2:119836043 | G | T | 0.2188 | 62795 |

**Supplementary Table 5: Sensitivity analysis of the effect of the methQTL SNPs on the association between the associated dmCpG and outcome levels in the Gambian children**

| **CpG** | **SNP** | **Outcome** | **Estimate (main EWAS)** | **95% CI (adjusted model)** | **pvalue** |
| --- | --- | --- | --- | --- | --- |
| cg00368636 | rs11977584 | SBP residual | 0.0400 | 0.0197, 0.0491 | 4.33x10^-6^ |
| cg00368636 | rs2672007 | SBP residual | 0.0400 | 0.0197, 0.0491 | 4.33x10^-6^ |
| cg00368636 | rs474341 | SBP residual | 0.0400 | 0.0197, 0.0491 | 4.33x10^-6^ |
| cg00368636 | rs517258 | SBP residual | 0.0400 | 0.0197, 0.0491 | 4.33x10^-6^ |
| cg00368636 | rs532409 | SBP residual | 0.0400 | 0.0197, 0.0491 | 4.33x10^-6^ |
| cg00368636 | rs537150 | SBP residual | 0.0400 | 0.0197, 0.0491 | 4.33x10^-6^ |
| cg00368636 | rs549970 | SBP residual | 0.0400 | 0.0197, 0.0491 | 4.33x10^-6^ |
| cg00368636 | rs589139 | SBP residual | 0.0400 | 0.0197, 0.0491 | 4.33x10^-6^ |
| cg00368636 | rs589171 | SBP residual | 0.0400 | 0.0197, 0.0491 | 4.33x10^-6^ |
| cg00368636 | rs590455 | SBP residual | 0.0400 | 0.0197, 0.0491 | 4.33x10^-6^ |
| cg00368636 | rs608809 | SBP residual | 0.0400 | 0.0197, 0.0491 | 4.33x10^-6^ |
| cg00368636 | rs635028 | SBP residual | 0.0400 | 0.0197, 0.0491 | 4.33x10^-6^ |
| cg00368636 | rs658606 | SBP residual | 0.0400 | 0.0197, 0.0491 | 4.33x10^-6^ |
| cg00368636 | rs666389 | SBP residual | 0.0400 | 0.0197, 0.0491 | 4.33x10^-6^ |
| cg00368636 | rs71562986 | SBP residual | 0.0400 | 0.0197, 0.0491 | 4.33x10^-6^ |
| cg00368636 | rs482461 | SBP residual | 0.0400 | 0.0198, 0.0492 | 4.29x10^-6^ |
| cg00368636 | GSA-rs7796960 | SBP residual | 0.0400 | 0.0171, 0.0475 | 3.05x10^-5^ |
| cg00368636 | rs10269825 | SBP residual | 0.0400 | 0.0173, 0.0492 | 4.28x10^-5^ |
| cg00368636 | rs4717245 | SBP residual | 0.0400 | 0.0173, 0.0492 | 4.28x10^-5^ |
| cg00368636 | rs9638427 | SBP residual | 0.0400 | 0.0173, 0.0492 | 4.28x10^-5^ |
| cg00368636 | rs10214965 | SBP residual | 0.0400 | 0.0174, 0.0493 | 4.03x10^-5^ |
| cg00368636 | rs10214971 | SBP residual | 0.0400 | 0.0174, 0.0493 | 4.03x10^-5^ |
| cg00368636 | rs10231956 | SBP residual | 0.0400 | 0.0174, 0.0493 | 4.03x10^-5^ |
| cg00368636 | rs3846968 | SBP residual | 0.0400 | 0.0176, 0.0493 | 3.61x10^-5^ |
| cg00368636 | rs3846969 | SBP residual | 0.0400 | 0.0176, 0.0493 | 3.61x10^-5^ |
| cg00368636 | rs10949984 | SBP residual | 0.0400 | 0.0202, 0.0509 | 5.80x10^-6^ |
| cg00368636 | rs35888355 | SBP residual | 0.0400 | 0.02, 0.0516 | 8.84x10^-6^ |
| cg00368636 | rs11772443 | SBP residual | 0.0400 | 0.0207, 0.0517 | 4.74x10^-6^ |
| cg00368636 | rs564679 | SBP residual | 0.0400 | 0.0167, 0.0535 | 1.85x10^-4^ |
| cg00368636 | rs34324184 | SBP residual | 0.0400 | 0.016, 0.0495 | 1.30x10^-4^ |
| cg00368636 | rs13221773 | SBP residual | 0.0400 | 0.0174, 0.0516 | 7.54x10^-5^ |
| cg00368636 | rs1352081 | SBP residual | 0.0400 | 0.0174, 0.0516 | 7.54x10^-5^ |
| cg00368636 | rs2200482 | SBP residual | 0.0400 | 0.0174, 0.0516 | 7.54x10^-5^ |
| cg00368636 | rs60621462 | SBP residual | 0.0400 | 0.0174, 0.0516 | 7.54x10^-5^ |
| cg00368636 | rs6952160 | SBP residual | 0.0400 | 0.0178, 0.0535 | 8.86x10^-5^ |
| cg00368636 | rs4620170 | SBP residual | 0.0400 | 0.0183, 0.0518 | 4.21x10^-5^ |
| cg00368636 | rs11486715 | SBP residual | 0.0400 | 0.0195, 0.0523 | 1.81x10^-5^ |
| cg00368636 | rs10267906 | SBP residual | 0.0400 | 0.0172, 0.0524 | 1.05x10^-4^ |
| cg00368636 | rs7804725 | SBP residual | 0.0400 | 0.0174, 0.0534 | 1.14x10^-4^ |
| cg00368636 | rs13244870 | SBP residual | 0.0400 | 0.0178, 0.0519 | 6.42x10^-5^ |
| cg00368636 | rs9638439 | SBP residual | 0.0400 | 0.0178, 0.0519 | 6.42x10^-5^ |
| cg00368636 | rs9638441 | SBP residual | 0.0400 | 0.0189, 0.052 | 2.75x10^-5^ |
| cg00368636 | rs34615570 | SBP residual | 0.0400 | 0.017, 0.0524 | 1.24x10^-4^ |
| cg00368636 | rs7796045 | SBP residual | 0.0400 | 0.016, 0.0521 | 2.14x10^-4^ |
| cg13135286 | rs74226968 | LDL-C residual | -4.8580 | -1.2438, 0.0336 | 6.34x10^-2^ |
| cg13135286 | rs75332983 | LDL-C residual | -4.8580 | -1.2438, 0.0336 | 6.34x10^-2^ |
| cg13135286 | rs116607690 | LDL-C residual | -4.8580 | -1.2303, 0.0759 | 8.32x10^-2^ |
| cg13135286 | rs139163124 | LDL-C residual | -4.8580 | -1.2303, 0.0759 | 8.32x10^-2^ |
| cg13135286 | rs114957083 | LDL-C residual | -4.8580 | -1.8851, -0.0505 | 3.87x10^-2^ |
| cg13135286 | rs484354 | LDL-C residual | -4.8580 | -2.6116, 0.1515 | 8.10x10^-2^ |
| cg13135286 | exm2260003 | LDL-C residual | -4.8580 | -2.4562, 0.1861 | 9.22x10^-2^ |
| cg13135286 | rs561672 | LDL-C residual | -4.8580 | -2.4556, 0.2341 | 1.05x10^-1^ |
| cg13135286 | rs614038 | LDL-C residual | -4.8580 | -2.4556, 0.2341 | 1.05x10^-1^ |
| cg13135286 | rs74145814 | LDL-C residual | -4.8580 | -9.3169, -1.4881 | 6.83x10^-3^ |
| cg13135286 | rs511513 | LDL-C residual | -4.8580 | -3.3183, 0.4018 | 1.24x10^-1^ |
| cg13135286 | rs6663401 | LDL-C residual | -4.8580 | -9.0194, -1.4353 | 6.90x10^-3^ |
| cg13135286 | rs6659203 | LDL-C residual | -4.8580 | -4.2255, 0.4761 | 1.18x10^-1^ |
| cg13135286 | rs140539580 | LDL-C residual | -4.8580 | -3.5911, -0.112 | 3.70x10^-2^ |
| cg13819288 | rs4849749 | LDL-C residual | -2.2590 | -4.028, -0.6754 | 5.97x10^-3^ |
| cg13819288 | rs36009069 | LDL-C residual | -2.2590 | -5.9451, -0.5446 | 1.85x10^-2^ |
| cg13819288 | rs75106351 | LDL-C residual | -2.2590 | -4.0112, -0.9225 | 1.74x10^-3^ |
| cg13819288 | rs116549068 | LDL-C residual | -2.2590 | -4.005, -0.9482 | 1.49x10^-3^ |
| cg13819288 | rs13410962 | LDL-C residual | -2.2590 | -4.005, -0.9482 | 1.49x10^-3^ |
| cg13819288 | rs1530136 | LDL-C residual | -2.2590 | -4.005, -0.9482 | 1.49x10^-3^ |
| cg13819288 | rs10202747 | LDL-C residual | -2.2590 | -5.5453, -1.0774 | 3.67x10^-3^ |
| cg13819288 | rs13395242 | LDL-C residual | -2.2590 | -5.5453, -1.0774 | 3.67x10^-3^ |
| cg13819288 | rs13425098 | LDL-C residual | -2.2590 | -5.5453, -1.0774 | 3.67x10^-3^ |
| cg13819288 | rs141807893 | LDL-C residual | -2.2590 | -5.5453, -1.0774 | 3.67x10^-3^ |
| cg13819288 | rs143070097 | LDL-C residual | -2.2590 | -5.5453, -1.0774 | 3.67x10^-3^ |
| cg13819288 | rs7562612 | LDL-C residual | -2.2590 | -5.5453, -1.0774 | 3.67x10^-3^ |
| cg13819288 | rs9308771 | LDL-C residual | -2.2590 | -5.5453, -1.0774 | 3.67x10^-3^ |
| cg13819288 | rs9308772 | LDL-C residual | -2.2590 | -5.5453, -1.0774 | 3.67x10^-3^ |
| cg13819288 | rs9308773 | LDL-C residual | -2.2590 | -5.5453, -1.0774 | 3.67x10^-3^ |
| cg13819288 | rs9308774 | LDL-C residual | -2.2590 | -5.5453, -1.0774 | 3.67x10^-3^ |
| cg13819288 | rs1439950 | LDL-C residual | -2.2590 | -4.8867, -0.7699 | 7.08x10^-3^ |
| cg13819288 | rs745322 | LDL-C residual | -2.2590 | -4.8867, -0.7699 | 7.08x10^-3^ |
| cg13819288 | rs111838684 | LDL-C residual | -2.2590 | -5.5565, -1.319 | 1.47x10^-3^ |
| cg13819288 | rs13409086 | LDL-C residual | -2.2590 | -5.5565, -1.319 | 1.47x10^-3^ |
| cg13819288 | rs330773 | LDL-C residual | -2.2590 | -4.4152, -1.0879 | 1.19x10^-3^ |

CpG-outcome effect sizes for methQTL-adjusted models are compared to those from the main EWAS without adjustment for methQTL. CpG-outcome effect sizes that are significantly reduced in adjustment models are highlighted in yellow.

methQTL-adjusted regression model: CpG ~ Physiological Outcome + methQTL_SNP + Sex + Age + PC1 + PC2 + PC3 + PC4 + PC5 + PC6 + PC7 + PC8 + PC9 + PC10

**Supplementary Table 6: Association of identified *cis*-methQTL SNPs with LDL-Cholesterol levels**

| **CpG** | **SNP** | **Outcome** | **Estimate** | **Std. Error** | **t value** | **Pr(>\|t\|)** | **FDR** |
| --- | --- | --- | --- | --- | --- | --- | --- |
| cg13135286 | rs116607690 | LDL | 0.2680 | 0.0753 | 3.5603 | 4.44E-04 | 0.0117 |
| cg13135286 | rs139163124 | LDL | 0.2680 | 0.0753 | 3.5603 | 4.44E-04 | 0.0117 |
| cg13135286 | rs6659203 | LDL | 0.2671 | 0.0709 | 3.7652 | 2.08E-04 | 0.0117 |
| cg13135286 | rs561672 | LDL | 0.2709 | 0.0831 | 3.2605 | 1.27E-03 | 0.0200 |
| cg13135286 | rs614038 | LDL | 0.2709 | 0.0831 | 3.2605 | 1.27E-03 | 0.0200 |
| cg13135286 | rs74226968 | LDL | 0.2258 | 0.0721 | 3.1323 | 1.94E-03 | 0.0219 |
| cg13135286 | rs75332983 | LDL | 0.2258 | 0.0721 | 3.1323 | 1.94E-03 | 0.0219 |

**Supplementary table 7: cg13135286 methQTL SNPs LD analysis**

#captured 7 of 7 alleles at r^2 >= 0.8

#captured 100 percent of alleles with mean r^2 of 0.956

#using 3 Tag SNPs in 3 tests.

| **Allele** | **Best Test** | **r^2 w/test** |  |
| --- | --- | --- | --- |
| rs6659203 | rs6659203 | 1 |  |
| rs75332983 | rs75332983 | 1 |  |
| rs74226968 | rs75332983 | 1 |  |
| rs139163124 | rs75332983 | 0.845 |  |
| rs116607690 | rs75332983 | 0.845 |  |
| rs614038 | rs614038 | 1 |  |
| rs561672 | rs614038 | 1 |  |
| **Test** | **Alleles Captured** | | |
| rs75332983 | rs74226968,rs75332983,rs116607690,rs139163124 | | |
| rs614038 | rs614038,rs561672 | | |
| rs6659203 | rs6659203 | | |

**Supplementary Table 8: Causal mediation analysis testing the hypothesis that methylation at cg13135286 mediates the association between 3 tagging methQTL SNPs and LDL-cholesterol levels.**

| **1. cg13135286 mediating the effect of rs75332983 on LDL-Cholesterol levels:** | | | | | |
| --- | --- | --- | --- | --- | --- |
| Causal Mediation Analysis | |  |  |  |  |
| Nonparametric Bootstrap Confidence Intervals with the Percentile Method | | | | | |
|  |  |  |  |  |  |
|  | Estimate | 95% CI Lower | 95% CI Upper | p-value |  |
| ACME | 0.699 | -0.179 | 1.61 | 0.1092 |  |
| ADE | -0.398 | -1.307 | 0.49 | 0.3472 |  |
| Total Effect | 0.302 | 0.102 | 0.5 | 0.0024 | ** |
| Prop. Mediated | 2.319 | -0.712 | 8.89 | 0.1116 |  |
|  |  |  |  |  |  |
| **2. cg13135286 mediating the effect of rs614038 on LDL-Cholesterol levels:** | | | | | |
| Causal Mediation Analysis | |  |  |  |  |
| Nonparametric Bootstrap Confidence Intervals with the Percentile Method | | | | | |
|  |  |  |  |  |  |
|  | Estimate | 95% CI Lower | 95% CI Upper | p-value |  |
| ACME | 0.1242 | -0.01 | 0.27 | 0.072 | . |
| ADE | 0.104 | -0.16 | 0.35 | 0.417 |  |
| Total Effect | 0.2282 | 0.0297 | 0.42 | 0.028 | * |
| Prop. Mediated | 0.5443 | -0.1392 | 3.22 | 0.098 | . |
|  |  |  |  |  |  |
| **3. cg13135286 mediating the effect of rs6659203 on LDL-Cholesterol levels:** | | | | | |
| Causal Mediation Analysis | |  |  |  |  |
| Nonparametric Bootstrap Confidence Intervals with the Percentile Method | | | | | |
|  |  |  |  |  |  |
|  | Estimate | 95% CI Lower | 95% CI Upper | p-value |  |
| ACME | 0.06649 | -0.00834 | 0.14 | 0.072 | . |
| ADE | 0.19121 | -0.00345 | 0.39 | 0.053 | . |
| Total Effect | 0.2577 | 0.05869 | 0.46 | 0.013 | * |
| Prop. Mediated | 0.25802 | -0.05368 | 0.92 | 0.082 | . |

**Supplementary figure titles and legends:**

**Supplementary figure1: Quantile-Quantile (QQ) plots for all the association analyses carried out for the Gambian cohort.**

Dotted line denotes the 95% confidence interval under the null hypothesis of no associations. Lambda is the genomic inflation factor. *denotes analyses with uncorrected lambda>1.2 that underwent Bacon correction for genomic inflation. Reported lambdas are those after Bacon correction. SBP=Systolic blood pressure; DBP=Diastolic blood pressure; PP=Pulse pressure; HOMA2-S=Insulin sensitivity; LDL-C=Low-density lipoprotein cholesterol; HDL-C=High-density lipoprotein cholesterol

**Supplementary figure 2: Quantile-Quantile (QQ) plots for all the association analyses carried out for the Indian cohort.**

Dotted line denotes the confidence interval band of the diagonal. Lambda is the genomic inflation factor. *denotes analyses with uncorrected lambda>1.2 that underwent Bacon correction for genomic inflation. Reported lambdas are those after Bacon correction. SBP=Systolic blood pressure; DBP=Diastolic blood pressure; PP=Pulse pressure; HOMA2-S=Insulin sensitivity; LDL-C=Low-density lipoprotein cholesterol; HDL-C=High-density lipoprotein cholesterol

**Supplementary Figure 3: MethQTL analysis for dmCpGs associated with LDL-Cholesterol levels in the Gambian cohort.**

(A) Association between genotype at 7 methQTL SNPs associated with cg13135286 and LDL-Cholesterol residual (mmol/l) in the Gambian children. (B) Linkage disequilibrium (LD) plot across the cg13135286 methQTL SNPs. The LD plot is based on the measure of D’, with dark grey diamonds indicating strong LD (D’>0.8) and light grey being uninformative (C) Correlation matrix of the 7 methQTL SNPs associated with cg13135286. LDL-Cholesterol=Low-density lipoprotein cholesterol; methQTL=Methylation Quantitative Trait Loci.

**Supplementary figures:**

**Supplementary figure 1**


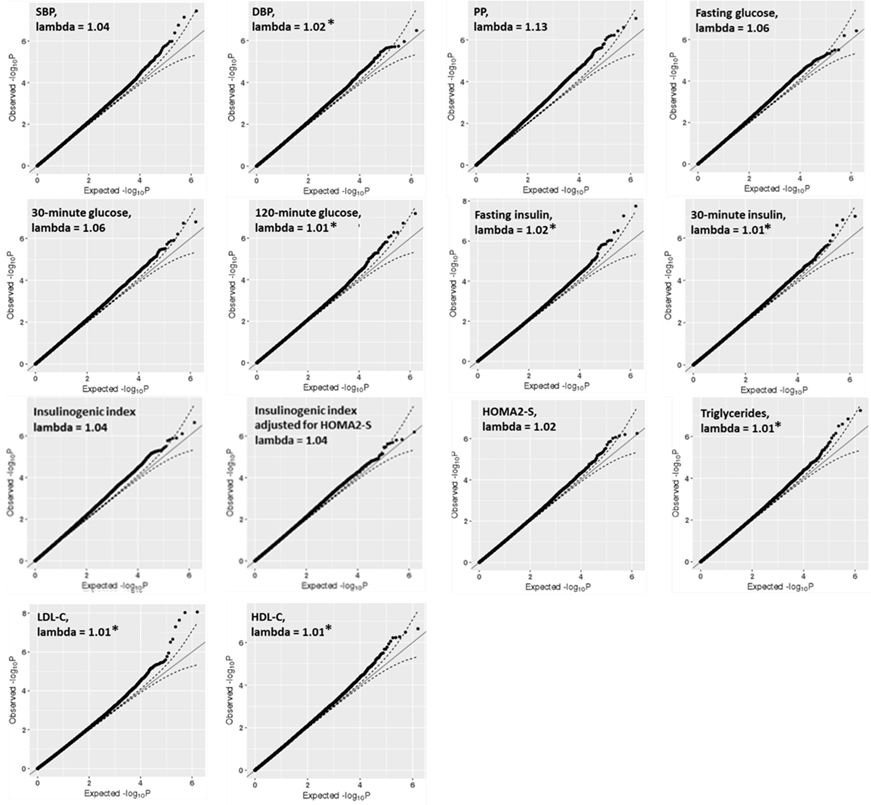


**Supplementary figure 2**


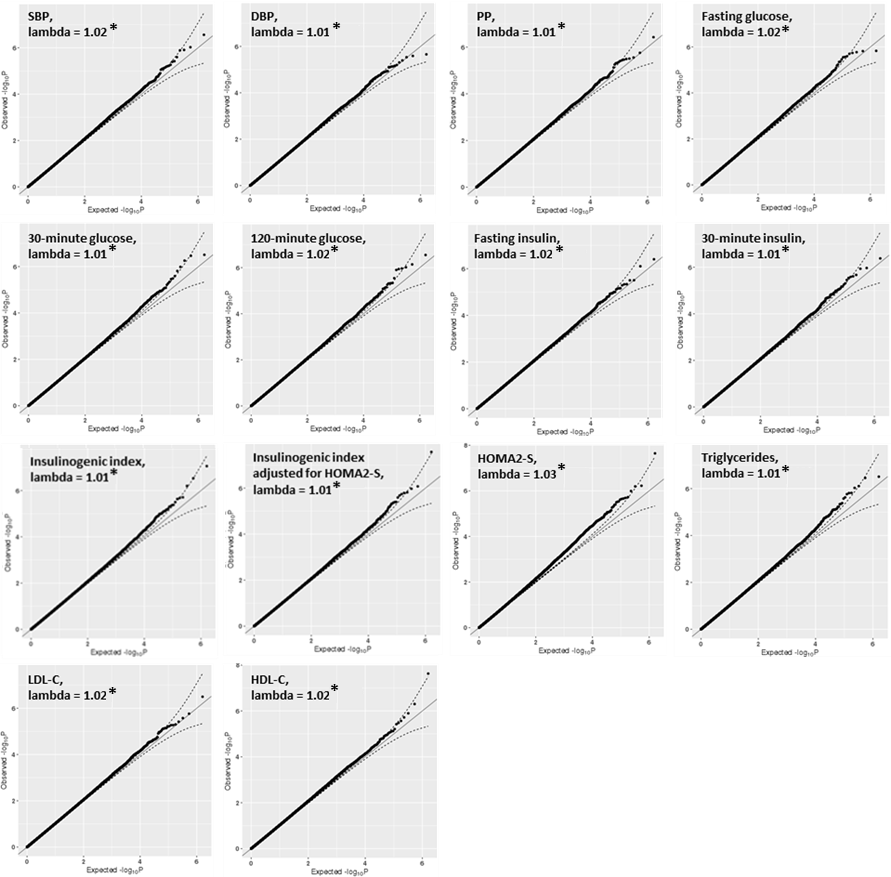


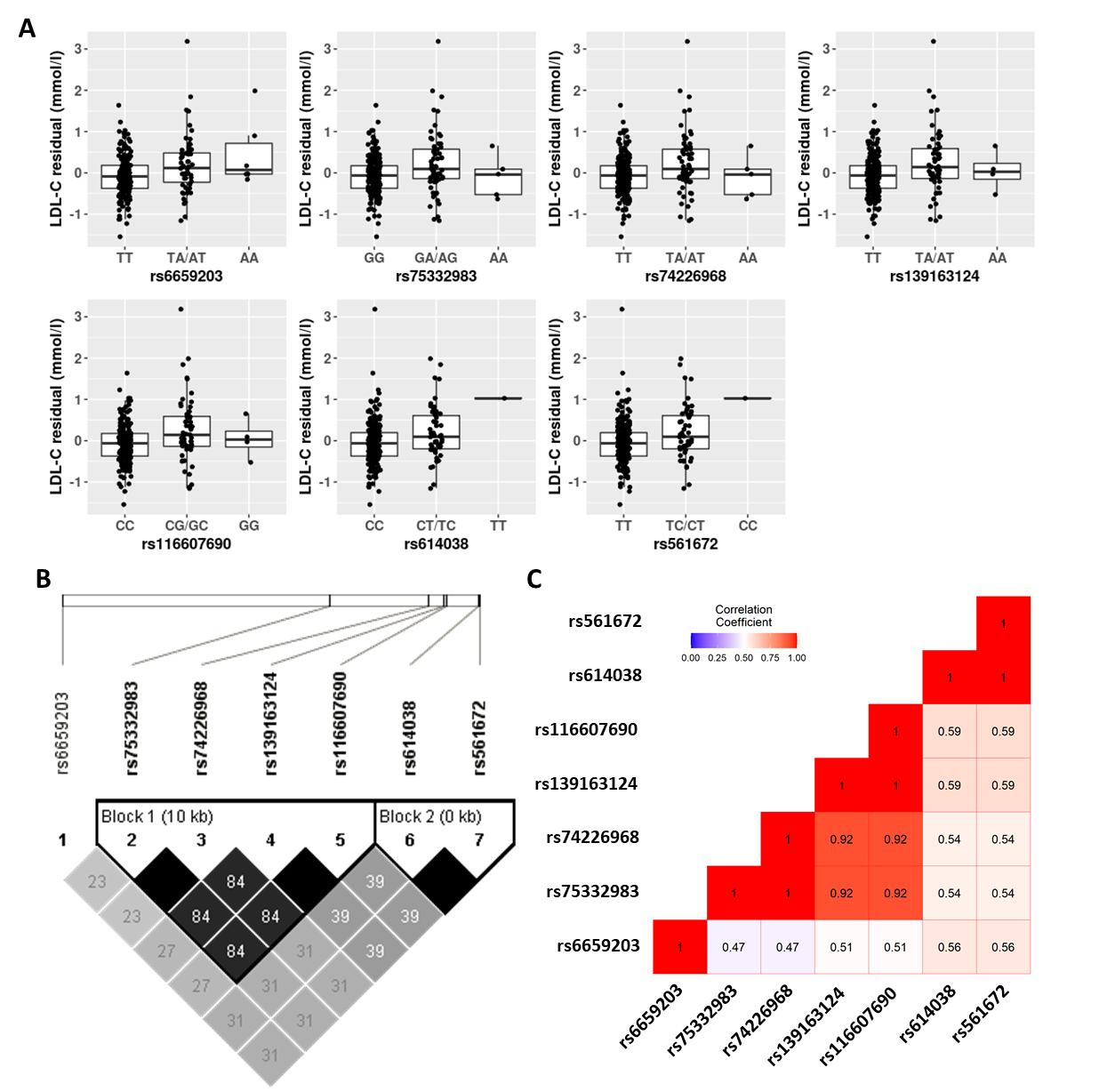
**Supplementary figure 3**
